# Accurate prediction of toxicity peptide and its function using multi-view tensor learning and latent semantic learning framework

**DOI:** 10.1093/bioinformatics/btaf489

**Published:** 2025-09-04

**Authors:** Ke Yan, Shutao Chen, Bin Liu, Hao Wu

**Affiliations:** School of Computer Science and Technology, Beijing Institute of Technology, Beijing 100081, China; Zhongguancun Academy, Beijing 100094, China; School of Computer Science and Technology, Beijing Institute of Technology, Beijing 100081, China; School of Computer Science and Technology, Beijing Institute of Technology, Beijing 100081, China; Zhongguancun Academy, Beijing 100094, China; SMBU-MSU-BIT Joint Laboratory on Bioinformatics and Engineering Biology, Shenzhen MSU-BIT University, Shenzhen, Guangdong 518172, China; School of Computer Science and Technology, Beijing Institute of Technology, Beijing 100081, China

## Abstract

**Motivation:**

Therapeutic peptide is an important ingredient in the treatment of various diseases and drug discovery. The toxicity of peptides is one of the major challenges in peptide drug therapy. With the abundance of therapeutic peptides generated in the post-genomics era, it is a challenge to promptly identify toxicity peptides using computational methods. Although several efforts have been made, few algorithms are designed to identify whether a query peptide exhibits toxicity. Considering the varied levels of biological activities, the toxicity peptides should be further classified into multi-functional peptides.

**Results:**

This study introduces a two-level predictor, ToxPre-2L, developed using the multi-view tensor learning and latent semantic learning framework. The proposed method utilized multi-label learning with feature induced labels to avoid the redundancy of information from each view. Then the multi-view tensor learning was employed to establish the latent semantic information among different views, while low-rank constraint learning was leveraged to exploit the correlation information among multi-labels. Finally, we constructed an updated toxicity peptide benchmark dataset to assess the effectiveness of the proposed method. Experimental results demonstrated that ToxPre-2L achieves a better performance than alternative computational methods in the prediction of toxicity peptides and their multi-functional types.

**Availability and implementation:**

The source code and data of ToxPre-2L can be accessed at http://bliulab.net/ToxPre-2L.

## 1 Introduction

Therapeutic peptide-based drugs have achieved an unprecedented surge of interest over the last decades, resulting in a robust development process. Therapeutic peptides are short chains with 50 or fewer amino acids and are pivotal in addressing a spectrum of pathological conditions within clinical treatment paradigms (Wei *et al.* 2021a, Gohil and Thirugnanasambandan 2021). Compared to small molecules, therapeutic peptides exhibit heightened biological activity and selectivity, resulting in a relatively lower incidence of side effects (Jin *et al.* 2024). Therefore, therapeutic peptides have unique characteristics to attribute to the therapeutic agents and drug development (Su *et al.* 2024).

Therapeutic peptides, considered as the category of biodrugs, have several inherent drawbacks, such as the issues of toxicity, immunogenicity, and stability (Wei *et al.* 2021b). Several strategies have been suggested to enhance the stability of the peptides, e.g. peptide cyclization. Similarly, several *in silico* tools are proposed to predict the immunogenicity properties of the peptides (Wei *et al.* 2018). However, the current state of peptide toxicity research reveals a paucity of proposed methodologies that adequately address the limitation, a critical factor essential for optimizing the therapeutic application. While peptides show considerable promise as therapeutic agents, their clinical translation faces significant challenges due to potential cytotoxicity, particularly their demonstrated hemolytic activity against red blood cells and other eukaryotic cell membranes.

The conventional machine learning methods for the prediction of toxicity peptides (TXP) contain two stages, including extracting the discriminative features to represent the peptides and the taxonomy-based predictors to distinguish the toxic peptides from the nontoxic ones(Fang *et al.* 2023). In the first stage, several hand-crafted features are designed to represent the inherent characteristics of peptide sequences, such as the kmer (Yan *et al.* 2022), Pse-AAC (Shen and Chou 2008), etc. In the second stage, several discriminative basic classifiers are utilized to predict the toxicity of peptides (Hearst *et al.* 1998, Qi 2012), such as linear regression (LR) (Weisberg 2005), etc. ToxinPred (Gupta *et al.* 2013) utilized the support vector machine (SVM) *in silico* method to predict the TXP, employing several sequence features, including the amino acid component (AAC) (Li *et al.* 2021), dipeptide composition (DC) (Li *et al.* 2021), etc. ClanTox (Naamati *et al.* 2009) extracted 545-D features to train a boosted stump classifier for animal toxins. ToxTeller (Wang and Sung 2024) employs physicochemical properties and sequence composition features, combined with four machine learning algorithms (LR, SVM, random forest (RF) (Lv *et al.* 2019), and XGBoost (Yang *et al.* 2021)), for TXP prediction. These methods utilized the conventional machine to make contribution to the TXP prediction. However, these two methods do not incorporate sequence-order or positional dependency information—a significant limitation for amino acid and nucleic acid sequence analysis (Wei *et al.* 2022).

Deep learning methods have found widespread application in TXP prediction, demonstrating a preference for generating high-latent features from sequences over conventional machine learning-based approaches, leveraging the limited peptide knowledge available (Wei *et al.* 2021b, Sreeraman *et al.* 2023, Wang *et al.* 2023a, Chen *et al.* 2025). Numerous predictors utilizing different deep learning frameworks have been proposed for therapeutic peptide prediction (Veltri *et al.* 2018, Wei *et al.* 2018, Zhang *et al.* 2021, Tang *et al.* 2022, Yan *et al.* 2022, Ispano *et al.* 2023, Qi and Zou 2023, Yan *et al.* 2023a,b, Ren *et al.* 2024), such as the attention mechanism (Vaswani *et al.* 2017, Su *et al.* 2024). ATSE (Wei *et al.* 2021b) and ToxIBTL (Wei *et al.* 2022) utilized the peptide evolutionary, structural and physicochemical information and deep learning framework to predict the TXP. However, ATSE relies on the position-specific scoring matrix (PSSM) for evolutionary information. This approach is time-consuming because PSSM construction requires PSI-BLAST (Altschul *et al.* 1997) searches across a large dataset. Pan *et al.* (2020) developed a deep learning-based approach named ToxDL, which employs convolutional neural networks to predict protein toxicity by leveraging both protein structural information and domain features. But this method is limited by its reliance on querying the UniProt database to obtain protein domain information for each target protein and its specificity to protein toxicity prediction rather than broader TXP assessment (Wei *et al.* 2022). ToxGIN (Yu *et al.* 2024) utilized the graph isomorphism networks to predict TXP. Although several previous methods have been proposed, those methods still have some drawbacks. (i) TXP represents a protein functional collection characterized by a diverse range of subgroups, such as anti-bacterial peptide (ABP) (Yu *et al.* 2021), anti-microbial peptide (AMP) (Javadpour *et al.* 1996), anti-cancer peptide (ACP) (Zhao *et al.* 2021), anti-fungal peptide (AFP) (de Ullivarri *et al.* 2020). This diversity suggests that the peptide toxicity within each subgroup may exhibit varying biological activities. (ii) The presented methods fail to address the crucial label correlations between different subgroups (Liu *et al.* 2024), limiting their performance in TXP prediction.

Multi-view learning frameworks integrate peptide sequence information from diverse data sources to extract more discriminative features than single-view approaches (Yan *et al.* 2019). Recent advances have expanded these frameworks to better model latent semantics through subspace learning, capturing shared and complementary information across views (Xiao *et al.* 2024). Notably, TPpred-ATMV (Yan *et al.* 2022) employed a multi-view tensor learning (MVTL) framework to predict therapeutic peptides. Building on these foundations, newer studies (Kang *et al.* 2025) have further optimized multi-view integration using deep representation learning, demonstrating improved generalizability in peptide function prediction. AmpHGT (He *et al.* 2025) leverages multi-view structural representations to construct heterogeneous graph networks and introduces an end-to-end deep learning architecture for AMP prediction.

Despite significant advancements in peptide toxicity prediction, current methods still exhibit limitations in classification accuracy, leading to a non-negligible rate of misclassified samples. Developing more precise predictive models is essential to enhance the reliability of toxicity assessments and strengthen confidence in the identification of TXP and their subtype classifications. In this work, a novel computational predictor, ToxPre-2L, was developed within a two-level framework to predict the TXP and their multi-functional types, such as anti-bacterial, anti-fungal, etc. To our knowledge, ToxPre-2L is the first computation predictor that adopts MVTL to predict TXPs and their multi-functional types. The first-level classifier utilizes the MVTL model to predict whether the query peptide is TXP. The second-level classifier utilizes the MVTL and binary relevance (BR) with the low-rank model to predict which multi-functional types the peptide belongs to if it has been identified as a TXP in the first level. We make several contributions as follows:

1. Our model employs a hybrid peptide encoding strategy that synergistically combines sequence-order information and physicochemical properties to capture both structural and functional determinants (Lemanov *et al.* 2003, Wei *et al.* 2022). The sequence-order features, particularly implemented through distance-based residue (DR) and distance pair (DP) representations, explicitly account for residue interactions at varying sequence intervals, thereby preserving critical spatial patterns. The pseudo amino acid composition (Pse-AAC) integrates intrinsic physicochemical attributes (e.g. hydrophobicity, hydrophilicity) with sequence composition, and *k*-mer measures the peptide composition information. Therefore, this multi-view encoding framework enables a comprehensive representation of peptides.
2. We employed a multi-view tensor learning framework to supervise feature learning, enabling the construction of an optimized latent subspace that effectively integrates complementary information from multiple feature views while maximizing the retention of discriminative information for TXP prediction.
3. The proposed framework employs a low-rank constraint to explicitly exploit higher-order label correlations across functional subgroups, effectively capturing latent label relationships and consequently boosting multi-label classification performance.
4. The proposed method supports interpretable insights into the latent semantic subspace. Furthermore, we have developed ToxPre-2L, a publicly accessible webserver available at http://bliulab.net/ToxPre-2L.

## 2 Materials and methods

### 2.1 Benchmark dataset

We construct a new toxicity dataset for TXP prediction. For the first-level binary classification task, we construct a benchmark dataset, which contains the positive sequences and negative sequences. The positive sequences sourced from our preceding work (Lv *et al.* 2023) are used to predict the TXP and the other therapeutic functional peptides. The positive sequences contain 2345 TXP sequences with a range of 10–50 residues, which are experimentally validated. For the negative nontoxic peptide (Non-TXP) sequences collection, the following steps were taken: (i) The peptides were sourced from the Swiss-prot dataset (Bairoch and Apweiler 1996) and the entries containing the related keywords were removed, including “Toxic,” “Toxin,” “KW-0020,” “KW-0800.” (ii) The Non-TXP with a length of 10–50 sequences were selected to keep the same distribution with the TXP sequences, and the Non-TXP with nonstandard residues were eliminated. (iii) The duplicated peptides that appeared from the previous dataset (Wei *et al.* 2021b) were removed. (iv) In order to reduce the homology bias and redundancy, the sequences with similarity exceeding 90% (Khosravian *et al.* 2013, Veltri *et al.* 2018, Burdukiewicz *et al.* 2020, Kavousi *et al.* 2020) within the nontoxic subset were filtered out using CD-HIT (Huang *et al.* 2010), resulting in 4125 Non-TXP sequences as the negative dataset. In order to construct the balanced dataset, we randomly selected 2345 Non-TXP sequences from the remaining negative dataset. Finally, we constructed a balanced dataset, where the number of TXP and Non-TXP sequences is the same. The first-level benchmark dataset is described by:


(1) $$S=S^{\mathrm{TXP}}\cup S^{\mathrm{Non}-\mathrm{TXP}}$$

where $S^{\mathrm{TXP}}$ is the TXP positive dataset. $S^{\mathrm{Non}-\mathrm{TXP}}$ is the Non-TXP negative dataset.

For the second-level multi-functional benchmark dataset, the positive TXP dataset can be further divided into nine functional categories, i.e.


(2) $$S^{\mathrm{TXP}}=S_{1}^{\mathrm{TXP}\;\mathrm{with}\;\mathrm{mono}-\mathrm{label}}\cup S_{2}^{\mathrm{ABP}}\cdots \cup S_{9}^{\mathrm{AVP}}$$

where subscripts 1,…,9 is the “TXP with mono-label”, “ABP”, “ACP”, “AMP”, “AFP”, “cell-penetrating peptide (CPP)”, “anti-parasitic peptide (APP)”, “drug delivery vehicle peptide (DDV)”, “anti-viral peptide (AVP)”, respectively. The statistical information of the second-level multi-functional dataset is illustrated in Supplementary Material 1, Fig. 1, available as supplementary data at *Bioinformatics* online. For parameters optimization and model evaluation, we employed 10-fold cross-validation strategy to roughly split the benchmark datasets S and $S^{\mathrm{TXP}}$ into the training, validation and test datasets with the ratio of 8:1:1.

### 2.2 Method overview

**Figure 1.**
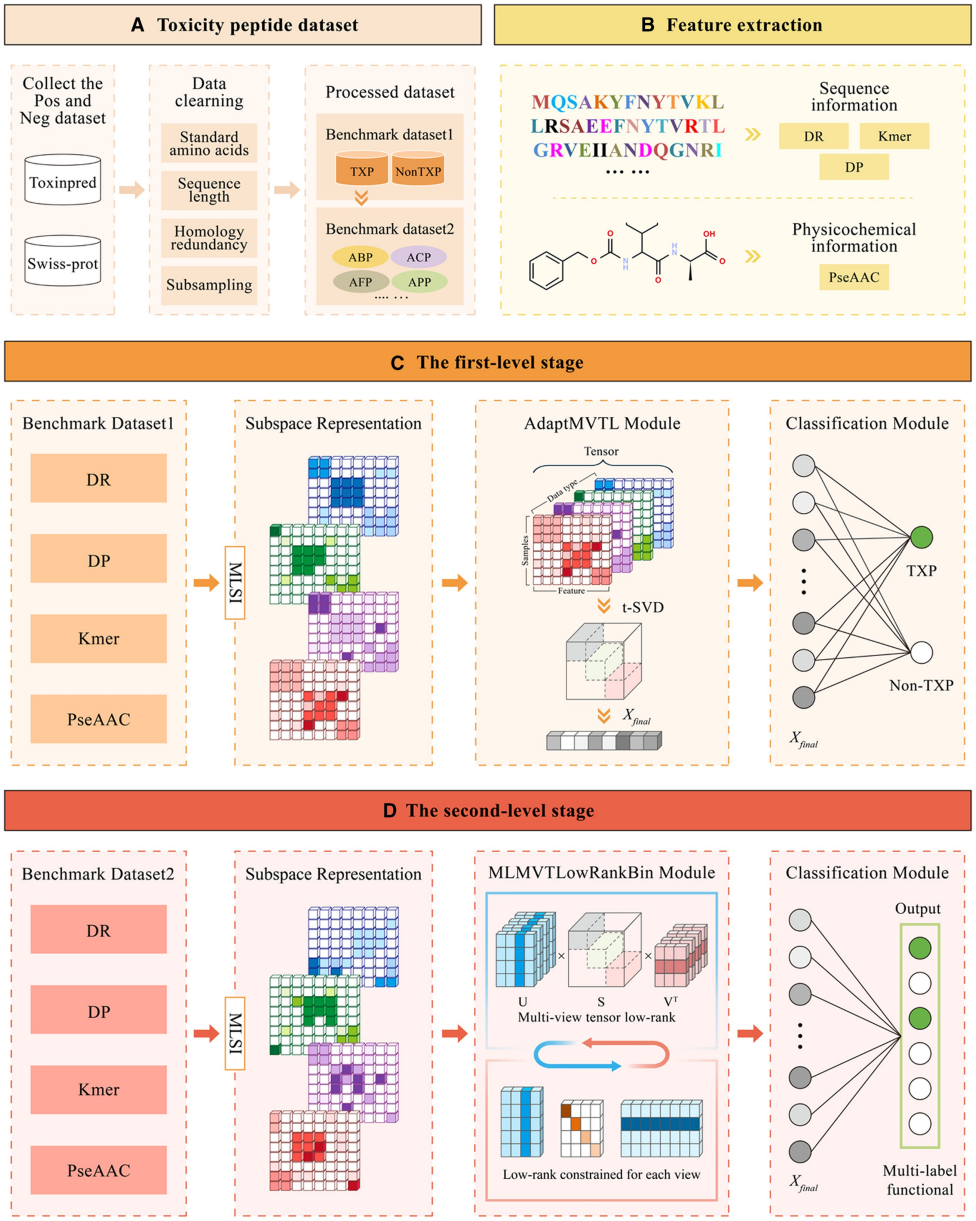
The framework of ToxPre-2L contains four stages. (A) The toxicity peptide dataset construction phase. (B) The feature extraction phase. The peptides are embedded by four feature-encoded methods that rely on sequential and physicochemical information. (C) The first-level stage. The input peptide sequences are first predicted by ToxPre-2L as TXP or Non-TXP. (D) The second-level stage. The predicted TXP sequences are further identified by ToxPre-2L as multi-label functional types.

ToxPre-2L consists of two-level framework to identify the TXP and their subfunctions. Initially, ToxPre-2L uses various biological tools to represent peptide sequences, extracting multi-view feature representations. Subsequently, ToxPre-2L proposes a two-level stages architecture based on the multi-view tensor learning and latent semantic learning framework. The first-level stage of ToxPre-2L detects whether a query peptide is a TXP or Non-TXP, while the second-level stage further determines TXP among nine functional attributes. The model of the first-level stage was trained with the benchmark dataset1 S [cf. Equation (1)] and the model of the second-level stage was trained with the benchmark dataset2 $S^{\mathrm{TXP}}$ [cf. Equation (2)]. Finally, the optimization algorithms of the ToxPre-2L are presented in Supplementary Material 2, available as supplementary data at *Bioinformatics* online, in detail. The model architecture is illustrated in Fig. 1.

### 2.3 Feature extraction methods

We utilized four feature extraction methods to encode the sequences, including the *k*-mer, DR, DP, and Pse-AAC. The *K*-mer (Khatun *et al.* 2020, Zulfiqar *et al.* 2024) feature considers the local sequence information by calculating the composition of subsequences with a fixed length *K*. The DR (Liu *et al.* 2014) feature calculates the composition of amino acid pairs between the *LG* spaces in the peptide. The Pse-AAC feature (Shen and Chou 2006) incorporates both amino acid composition and physicochemical properties, while the DP feature (Liu *et al.* 2014, Zhu *et al.* 2023, Zou *et al.* 2023) captures pairwise residue interactions by combining spatial distance constraints with sequence-order information providing complementary representations of peptide sequences. The detailed information of four features is described in Supplementary Material 1, available as supplementary data at *Bioinformatics* online. The above four features were extracted by Bioseq-Analysis 2.0 (Liu *et al.* 2019, Li *et al.* 2021) with default parameters.

### 2.4 Architecture of ToxPre-2L

The training dataset contains n peptides ${\left\{x_{i},y_{i}\right\}}_{i=1}^{n}$, where $x_{i}\in R^{m}$ is the feature of the ith peptide sequence and $y_{i}\in {\left\{-1,1\right\}}^{l}$ represents the label vector corresponding to $x_{i}$. l denotes the total number of potential labels. $y_{is}=1$ (or −1) indicates the ith peptide sequence’s sth label (s∈[1,L]) is relevant (or irrelevant). The goals of the first-level stage and the second-level stage of the ToxPre-2L are to learn the binary labels classifier (L=2) and the multi-label classifier (L=9), respectively.

Suppose that n training peptide sequences and r test peptide sequences are encoded in D views. Let ${\tilde{X}}_{tr}=[x_{\mathrm{tr}}^{1,(d)};\ldots ;x_{\mathrm{tr}}^{n,(d)}]\in R^{n\times m_{d}}$ be the training peptides from the dth(d∈[1,…, D]) view, where $x_{\mathrm{tr}}^{i,(d)}\in R^{m_{d}}$ is a $m_{d}$-dimensional real-valued training peptide sequence vector from the dth view. $Y=[y^{1},\ldots ,y^{n}]\in R^{n\times l}$ is the label matrix of the ${\tilde{X}}_{\mathrm{tr}}$ peptides. The r test peptide sequences from the dth view are represented as ${\tilde{X}}_{\mathrm{tt}}=[x_{\mathrm{ts}}^{1,(d)};\cdots ;x_{\mathrm{ts}}^{r,(d)}]\in R^{r\times m_{d}}$.

To extract the effective information of each view feature, we utilized the multi-label information latent semantic index (MLSI) (Kai *et al.* 2005) strategy to reduce the dimensionality of the feature. MLSI selected the best feature subset to represent the intricate information structure inherent in multi-label features. The MLSI utilized the kernel function to map the input features into high-dimensional non-linear space. $k_{x}(x_{i},x_{j})$ (radial basis function) represents the inner product as


(3) $$k_{x}(x_{i},x_{j})=\exp(-\theta {\left\|x_{i}-x_{j}\right\|}^{2})$$

where θ is the bandwidth. Subsequently, MLSI selected the subspace features that collectively represent the intricate information structure inherent in each view feature representation. Therefore, MLSI focused on the relevant features and discovered the latent semantic space information with each view feature. We selected each view latent semantic feature subset $X_{\mathrm{tr}}\in R^{n\times s}$ and $X_{\mathrm{tt}}\in R^{r\times s}$ from source multi-view information ${\tilde{X}}_{\mathrm{tr}}$ and ${\tilde{X}}_{\mathrm{tt}}$ by using the MLSI.

#### 2.4.1 First-level stage: the peptide toxicity sub-predictor by adaptive weighted MVTL (AdaptMVTL) model

Motivated by the MVTL framework, we embedded the peptides from multi-view different features into the common latent space and captured the correlation between multi-view features in the following model:


(4) $$\underset{P^{\left(d\right)},z}{\min}\sum_{d=1}^{D}{\left\|X_{\mathrm{tr}}^{\left(d\right)}P^{\left(d\right)}-Y\right\|}_{F}^{2}+\lambda_{1}\sum_{d=1}^{D}{\left\|P^{\left(d\right)}\right\|}_{2}^{2}+\lambda_{2}{\left\|z\right\|}_{⊛}$$

where $z={\left\{X_{\mathrm{tr}}^{\left(d\right)}P^{\left(d\right)}\right\}}_{d=1}^{D}$, ${\left\|z\right\|}_{⊛}$ denotes the tensor nuclear norm. $\lambda_{1}>0$ and $\lambda_{2}>0$ are the tradeoff factors. To capture crucial information across different views, the proposed method utilized the t-SVD (Wu *et al.* 2019) to capture the largest singular values of the slice of the *f*-diagonal tensor.

Furthermore, we utilized non-negative automatic weights, denoted as $a^{\left(d\right)}(a^{\left(d\right)}\geq 0)$, corresponding to the dth view. A greater weight $a^{\left(d\right)}$ indicates a more significant role of the corresponding view feature in multi-view learning. Accordingly, the final sub-predictor function AdaptMVTL turns out to be


$$\underset{a^{\left(d\right)},\;P^{\left(d\right)},z}{\min}\sum_{d=1}^{D}{a^{\left(d\right)}\|X_{\mathrm{tr}}^{\left(d\right)}P^{\left(d\right)}-Y\|}_{F}^{2}+\lambda_{1}\sum_{d=1}^{D}{\left\|P^{\left(d\right)}\right\|}_{2}^{2}+\lambda_{2}{\left\|z\right\|}_{⊛}+\gamma {\left\|a\right\|}_{2}^{2}$$

(5) $$s.t.\;a^{\left(d\right)}\geq 0,\sum_{d=1}^{D}a^{\left(d\right)}=1$$

where γ>0 is a penalty parameter and $a=[a^{\left(1\right)},a^{\left(2\right)},\ldots ,a^{\left(D\right)}]$ is the weight vector, where $a^{\left(d\right)}$ is the relative weight of the dth view.

When in prediction for a test peptide sequence $x_{\mathrm{tt}}^{\left(d\right)}$ from *D* views, we first achieve the $f=\sum_{d=1}^{D}a^{\left(d\right)}x_{\mathrm{tt}}^{\left(d\right)}P^{\left(d\right)}(f\in R^{l})$, then get the predicted binary label result


(6) $$y_{\mathrm{tt}}=\mathrm{argmax}(f)$$

Then the model obtains l scores corresponding to the TXP and Non-TXP and each score is accumulated from *D* views. A large score denotes the test peptide sequence $x_{\mathrm{tt}}$ is more likely to belong to the corresponding label. The optimization solution of the AdaptMVTL model is presented in Supplementary Material 2, available as supplementary data at *Bioinformatics* online.

In summary, the first stage sub-predictor AdaptMVTL of ToxPre-2L has the following merits: (i) our model constructs the common representation among multi-view features by using latent semantic learning; and (ii) our model utilizes the MVTL to capture the strong correlation between different views.

#### 2.4.2 Second-level stage: a unified TXP’s functional types sub-predictor for multi-label multi-view tensor learning with low-rank and BR (MLMVTLowRankBin) model

To capture the diverse characteristics of multiple functional types, the proposed method constructs *L* binary predictors using BR. This BR step is then integrated into the AdaptMVTL framework, which can be formulated as follows:


(7) mina(d), P(d),z,t∑d=1Da(d)‖Xtr(d)P(d)-Y‖F2+λ1∑d=1D‖P(d)‖22+λ2‖z‖⊛+γ‖a‖22+∑d=1D∑i=1n∑j=1Lj∈yi+pj,(d),xtri,(d)≤t(xtri,(d))+j∈yi-pj,(d),xtri,(d)≥t(xtri,(d))s.t. a(d)≥0,∑d=1Da(d)=1

where $y_{i}^{+}$ ($y_{i}^{-}$) represents the index set of relevant (irrelevant) labels linked to the peptide sequence $x_{\mathrm{tr}}^{i}$. $t(x_{\mathrm{tr}}^{i,(d)})$ denotes the ideal thresholding value. $⟦g⟧$ equals 1 when the g holds, and 0 otherwise.

Due to the threshold $t(x_{\mathrm{tr}}^{i,(d)})$ being associated with the parameter matrix $P^{\left(d\right)}$ and the corresponding sequence $x_{\mathrm{tr}}^{i,(d)}$, the $t(x_{\mathrm{tr}}^{i,(d)})$ is difficult to optimization. Inspired by the RBRL (Wu *et al.* 2020), we fixed the threshold values $t(x_{\mathrm{tr}}^{i,(d)})=0(i\in [1,n])$ for n peptide sequences for simplicity. Moreover, we approximated the thresholding 0–1 loss function with the surrogate least squared hinge loss $\mathrm{loss}(y,f(x))=\max{\left(0,1-yf(x)\right)}^{2}={\left({\left|1-yf(x)\right|}_{+}\right)}^{2}$ (Wu *et al.* 2020). Therefore, the problem is transformed as follows:


(8) mina(d), P(d),z∑d=1Da(d)‖Xtr(d)P(d)-Y‖F2+∑d=1D12‖(|E-Yȯ(Xtr(d)P(d))|+)2‖1+λ1∑d=1D‖P(d)‖22+λ2‖z‖⊛+γ‖a‖22s.t. a(d)≥0,∑d=1Da(d)=1 

where $E\in R^{n\times l}$ consists entirely of elements equal to 1. The second part of our proposed method introduces a nonlinear loss term that specifically penalizes under-prediction, ensuring more accurate alignment between the predicted labels and the true labels for each individual label.

Furthermore, we utilized a low-rank constraint on $P^{\left(d\right)}$ to harness the correlations among high-order labels. The final object function MLMVTLowRankBin turns out to be


(9) mina(d), P(d),z∑d=1Da(d)‖Xtr(d)P(d)-Y‖F2+∑d=1D12‖(|E-Yȯ(Xtr(d)P(d))|+)2‖1+λ1∑d=1D‖P(d)‖22+λ2‖z‖⊛+λ3∑d=1D‖P(d)‖*+γ‖a‖22s.t. a(d)≥0,∑d=1Da(d)=1 

Once we obtained the transformation matrix $P^{\left(d\right)}$, the multi-label TXP’s functional types $y_{\mathrm{tt}}$ of the test peptide sequences $x_{\mathrm{tt}}^{\left(d\right)}$ with *D* views is calculated as follows:


(10) $$y_{tt}=\mathrm{sign}\left(⟦\sum_{d=1}^{D}a^{\left(d\right)}x_{tt}^{\left(d\right)}P^{\left(d\right)}>0⟧\right)$$

where sign(x) is 1 when x>0 and −1 otherwise. The optimization solution of the MLMVTLowRankBin model is presented in Supplementary Material 2, available as supplementary data at *Bioinformatics* online.

Therefore, we proposed the MLMVTLowRankBin framework based on the elastic net regularized (Zhang *et al.* 2017) for TXP’s multi-functional types classification. In summary, the second stage sub-predictor MLMVTLowRankBin of ToxPre-2L has the following merits: (i) our model integrated a thresholding step into the AdaptMVTL framework by leveraging the BR approach, designed to achieve optimal data fitting and effectively address under-prediction; and (ii) our model utilized the trace norm to harness the correlations among high-order labels across diverse TXP’s multi-functional types. This enhancement is particularly crucial for improving performance in the multi-functional classification of TXP.

### 2.5 Performance evaluation

In this study, the 10 cross-validation strategy was used to assess the performance of ToxPre-2L. ToxPre-2L is a two-level stage predictor. The first-level stage of ToxPre-2L, which is dedicated to the classification of a test peptide as a TXP or Non-TXP, falls within the domain of single-label classification. The five metrics are commonly employed for assessing the efficacy of the single-label predictor, including the accuracy (ACC), sensitivity (SN), specificity (SP), Mathew’s correlation coefficient (MCC) (Chicco and Jurman 2020, Zhang *et al.* 2024), and area under curve (AUC). The AUC represents the area under the receiver operating characteristic (ROC) curve, evaluating a model’s ability to distinguish between classes (Bradley 1997, Tang *et al.* 2022, Zhu *et al.* 2023, Xie *et al.* 2024, Yan *et al.* 2024, Zhang *et al.* 2024).


(11) $$\{\begin{array}{c} \begin{array}{c} SN=\frac{\mathrm{TP}}{\mathrm{TP}+\mathrm{FN}}\\ SP=\frac{\mathrm{TN}}{\mathrm{FP}+\mathrm{TN}} \end{array}\\ \begin{array}{c} A\mathrm{CC}=\frac{\mathrm{TP}+\mathrm{TN}}{\mathrm{TP}+\mathrm{TN}+\mathrm{FN}+\mathrm{FP}} \end{array}\\ M\mathrm{CC}=\frac{\mathrm{TP}*\mathrm{TN}-\mathrm{FP}*\mathrm{FN}}{\sqrt{\left(\mathrm{TP}+\mathrm{FN})(\mathrm{TP}+\mathrm{FP})(\mathrm{TN}+\mathrm{FP})(\mathrm{TN}+\mathrm{FN}\right)}} \end{array}$$

where TP is the true positive, TN is the true negative, FN is the false negative, and FP is the false positive.

For the second-level stage, identifying a query TXP among nine functional types [cf. Equation (2)] falls within the domain of multi-label classification. Then we utilize six metrics, including hamming loss (HL), average precision (AP), coverage (CV), and accuracy (ACC). Assume that $\left\{(x_{i},D_{i})i\in [1,N]\right\}$ is the test set, $H_{i}$ is the predicted label of the test sequence $x_{i}$, $D_{i}$ is the true label of the test sequence $x_{i}$, and $D_{i}=\{l_{1},\ldots ,l_{q}\}.\;q$ is the number of the category label


(12) $$\{\begin{array}{c} \begin{array}{c} HL=\frac{1}{N}\sum_{i=1}^{N}\left(\frac{\left\|H_{i}\cup D_{i}\|-\|H_{i}\cap D_{i}\right\|}{q}\right) \end{array}\\ \begin{array}{c} AP=\frac{1}{N}\;\sum_{i=1}^{N}\left(\frac{1}{\left\|D_{i}\right\|}\sum_{l_{j},l_{k}\in D_{i}}\frac{\left\|\left\{l_{j}|\mathrm{rank}(x_{i},l_{j})\leq \mathrm{rank}(x_{i},l_{k})\right\}\right\|}{\mathrm{rank}(x_{i},l_{k})}\right)\\ CV=\frac{1}{N}\sum_{i=1}^{N}\left(\frac{\left\|H_{i}\cap D_{i}\right\|}{\left\|D_{i}\right\|}\right) \end{array}\\ A\mathrm{CC}=\frac{1}{N}\sum_{i=1}^{N}\left(\frac{\left\|H_{i}\cap D_{i}\right\|}{\left\|H_{i}\cup D_{i}\right\|}\right) \end{array}$$

where ∪ is the union of the labels in the two label vectors, ∩ is the intersection of the labels in the two label vectors. ‖‖ is the number of 1s in the vector.

## 3 Results and discussion

### 3.1 Comparing with existing predictors in identifying TXP and Non-TXP on the benchmark dataset S

**Figure 2.**
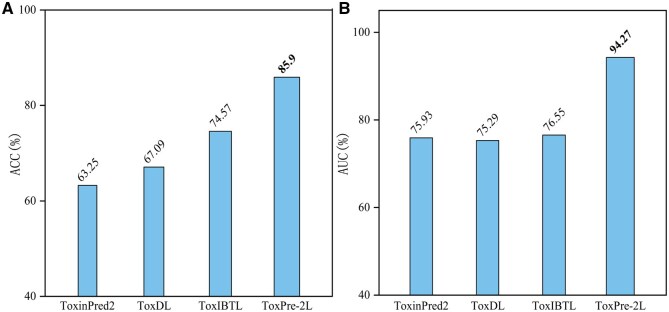
The prediction results of ToxPre-2L and other methods in terms of ACC and AUC metrics on the benchmark dataset S. The different methods are used to identify TXP and Non-TXP. The results of the compared methods were obtained through the stand-alone packages reported in Pan *et al.* (2020), Sharma *et al.* (2022), and Wei *et al.* (2022).

In this section, we conducted a comparative assessment of the performance of the proposed method against several state-of-the-art approaches in the discrimination of TXP and Non-TXP peptides. The single-label prediction performance achieved by the first-level stage of ToxPre-2L and the other compared methods for identifying TXP and Non-TXP in the benchmark dataset S is measured by the six metrics [cf. Equation (11)]. The compared methods include ToxinPred2 (Sharma *et al.* 2022), ToxDL (Pan *et al.* 2020), and ToxIBTL (Wei *et al.* 2022). The predicted results of different methods are shown in Fig. 2 and Supplementary Material 1, Fig. 2, available as supplementary data at *Bioinformatics* online, via 10-fold cross-validation on the benchmark dataset S. These parameters of the first-level stage subpredictor AdaptMVTL of ToxPre-2L were optimized on the validation set, which is dependent with the training set and test set. For fair comparison, we strictly adhered to the parameter settings recommended in the respective original papers of all compared methods. The hyper-parameter sets are documented in Supplementary Material 1, Table 1, available as supplementary data at *Bioinformatics* online, with corresponding literature citations. Based on the observations from Fig. 2 and Supplementary Material 1, Fig. 2, available as supplementary data at *Bioinformatics* online, it is evident that ToxPre-2L demonstrates superior performance compared to all other methods considered, exhibiting enhancements ranging from 11.33% to 22.65% in terms of AUC and ACC, and improvements spanning from 0.11 to 0.58 in MCC and *F*1 scores.

The results above discussed demonstrated that the proposed method utilized more discriminative and effective TXP specific features for distinguishing both TXP and Non-TXP peptide sequences. There are two reasons for the outstanding performance of ToxPre-2L. (i) Compared with the machine learning methods based on the hand-crafted features, which are constructed through the knowledge of peptides, the proposed method not only automatically extracts the latent semantic information from each view based on the MLSI method, but also constructs the common subspace information shared with the multiple views, which is critical for the sequence analysis. (ii) Compared with the ToxIBTL and its variants, which capture the high-latent features of the peptide sequences through the deep learning framework, the proposed method adopts tensor learning to learn the correlation information among different views that interfere with the TXP prediction. Therefore, ToxPre-2L combines the MLSI feature extraction strategy and tensor learning strategy to achieve optimal performance.

### 3.2 Comparing with the multi-label classification algorithms in prediction TXP’s functional types on the benchmark dataset $S^{\mathrm{TXP}}$

In this section, we assessed the multi-label prediction performance of the second-level stage sub-predictor of ToxPre-2L on the benchmark dataset $S^{\mathrm{TXP}}$. To our knowledge, ToxPre-2L is the first predictor in identifying the multi-functional types of TXP with various subgroup. Then we take several widely used multi-label predictors and multi-view predictors as comparison, including multi-instance multi-label learning *K*-nearest neighbor (MIML-KNN) (Zhang 2010), multi-instance multi-label learning radial basis function (MIML-RBF) (Zhang and Wang 2009), multi-label *K*-nearest neighbor (ML-KNN) (Zhang and Zhou 2007), Rank-SVM (Tayal *et al.* 2018, Wang *et al.* 2023b), multi-label with label-specific features (LIFT) (Zhang and Wu 2015), ETFC (Fan *et al.* 2023), IMFP (Luo *et al.* 2024), TPpred-LE (Lv *et al.* 2023), and TPpred-SC (Yan *et al.* 2024). The predictive outcomes for four metrics [cf. Equation (12)] are presented in Table 1 via 10-fold cross-validation strategy, demonstrating the superior performance of the proposed method compared to the alternative methods across these four metrics. All deep learning-based comparison methods (iMFP-LG, TPpred-LE, and TPpred-SC) were implemented using their original published hyper-parameter configurations. The hyper-parameter specifications are provided in Supplementary Material 1, Table 2, available as supplementary data at *Bioinformatics* online.

**Table 1.** Performance evaluation of methods using 10-fold cross-validation on the benchmark dataset $S^{\mathrm{TXP}}$.^a^

Method	Acc↑^a^	HL↓^a^	AP↑^a^	CV↓^a^
MIML-KNN	0.77	0.08	**0.98**	2.4
MIML-RBF	0.80	0.07	0.90	2.05
ML-KNN	0.81	0.07	**0.98**	2.03
RankSVM	0.58	0.12	0.94	1.7
LIFT	0.77	0.08	**0.98**	2.38
ETFC	0.62	0.09	0.71	2.64
IMFP	0.60	0.10	0.70	2.62
TPpred-LE	0.55	0.09	0.68	2.56
TPpred-SC	0.57	0.10	0.71	2.60
ToxPre-2L	**0.86**	**0.06**	0.94	**1.65**

a An upward arrow (↑) denotes that a higher metric value indicates superior performance, whereas a downward arrow (↓) signifies that a lower metric value corresponds to enhanced performance. The bold values represent the best results.

These results demonstrated that the ToxPre-2L can achieve more accurate and effective correlation information of labels between different Toxic’s functional types. In contrast to first-order strategy (Zhang and Zhou 2014) like ML-KNN, ToxPre-2L demonstrates superior performance concerning classification-based metrics (e.g. ACC, HL) primarily due to its incorporation of a low-rank constraint term. This term enables ToxPre-2L to effectively leverage label correlations across various function types, thereby enhancing its predictive capabilities in multi-functional prediction tasks. In contrast to the second-order strategy (Zhang and Zhou 2014) and multi-label therapeutic peptide predictions, including Rank-SVM, ETFC, iMFP-LG, TPpred-LE, and TPpred-SC, ToxPre-2L attains superior performance across classification-based metrics (i.e. ACC, HL) and ranking-based metrics (i.e. CV and AP). This is primarily attributed to the BR method, which addresses the low prediction problem caused by the imbalanced long-tail distribution of different subgroup function types of TXP. The proposed method places greater emphasis on under-predicted labels during the model optimization process by leveraging the BR method, thereby improving the prediction for these labels and ultimately reducing under-prediction. Therefore, the optimal performance of ToxPre-2L is achieved by integrating the BR into the MVTL framework, allowing for the training of the model in a single step. Additionally, the utilization of the low-rank constraint enables the exploitation of label correlations across diverse functional types of TXP, further enhancing the model’s effectiveness.

### 3.3 Analysis the latent semantic information

**Figure 3.**
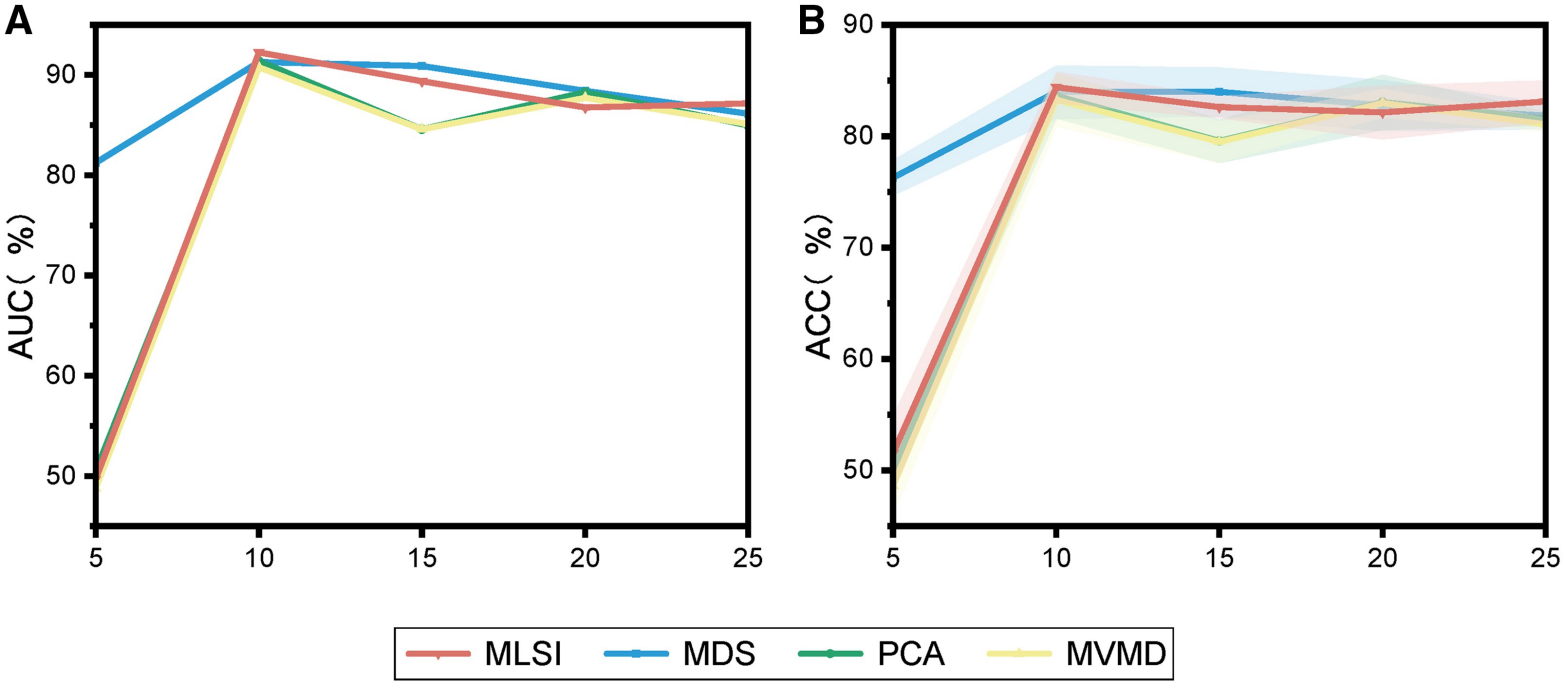
Comparison results based on different feature selection algorithms on benchmark dataset S. The error bars represent the mean ± standard deviation from the cross-validation experiments. When the 10-dimensional feature subset of each view is obtained by MLSI, the results of AUC and ACC achieve the best.

ToxPre-2L utilizes the MLSI to capture the latent semantic information from each view feature. MLSI considers the unique information posed by single-label classification and reduces the spatial dimensions by eliminating irrelevant features. Then we utilized several feature selection compared methods to evaluate the performance of MLSI, including principal component analysis (PCA) (Wright *et al.* 2009), MDDM (Zhang and Zhou 2010), MDS (Anowar *et al.* 2021). The dimension of the four feature extraction methods is taken from 5 to 25 with an interval of 5. Through 10-fold cross-validation, the feature subset constructed by each method is fed into AdaptMVTL. The results of four feature methods on the benchmark dataset S are shown in Fig. 3.

The results demonstrated that the MLSI algorithm achieves optimal prediction performance across all evaluation metrics when the dimension is set to 10. The comparative analysis reveals that the control methods (PCA, MVMD, MDS) exhibit notable instability across different feature dimensions, as evidenced by their larger standard deviations in both ACC and AUC metrics. For instance, PCA shows significant fluctuations in ACC (±2.47 SD at dimension 20) and AUC (±0.04 SD at dimension 5), likely due to its linearity assumption failing to capture complex data structures. MVMD demonstrates parameter sensitivity, with unstable performance in low dimensions (ACC: ±2.57 SD at dimension 5) and inconsistent AUC trends. While MDS achieves high ACC at dimension 5 (76.29 ± 1.6), its performance varies substantially (±2.4 SD at dimension 10), reflecting sensitivity to distance metrics and outliers. In contrast, MLSI maintains consistently lower variability (e.g. AUC ±0.02 SD vs. PCA’s ±0.04 SD), underscoring its robustness through adaptive feature integration and noise resistance. The MLSI takes into consideration the pertinent information associated with the interrelationships among labels and captures the latent semantic information to remove the redundant information in each view features, which greatly improve the prediction performance. This instability in control methods highlights their limitations in handling data variability, whereas MLSI’s stability validates its reliability for practical applications. Therefore, we utilize the MLSI as feature selection algorithm to capture the latent semantic information.

### 3.4 Interpretability of ToxPre-2L

**Figure 4.**
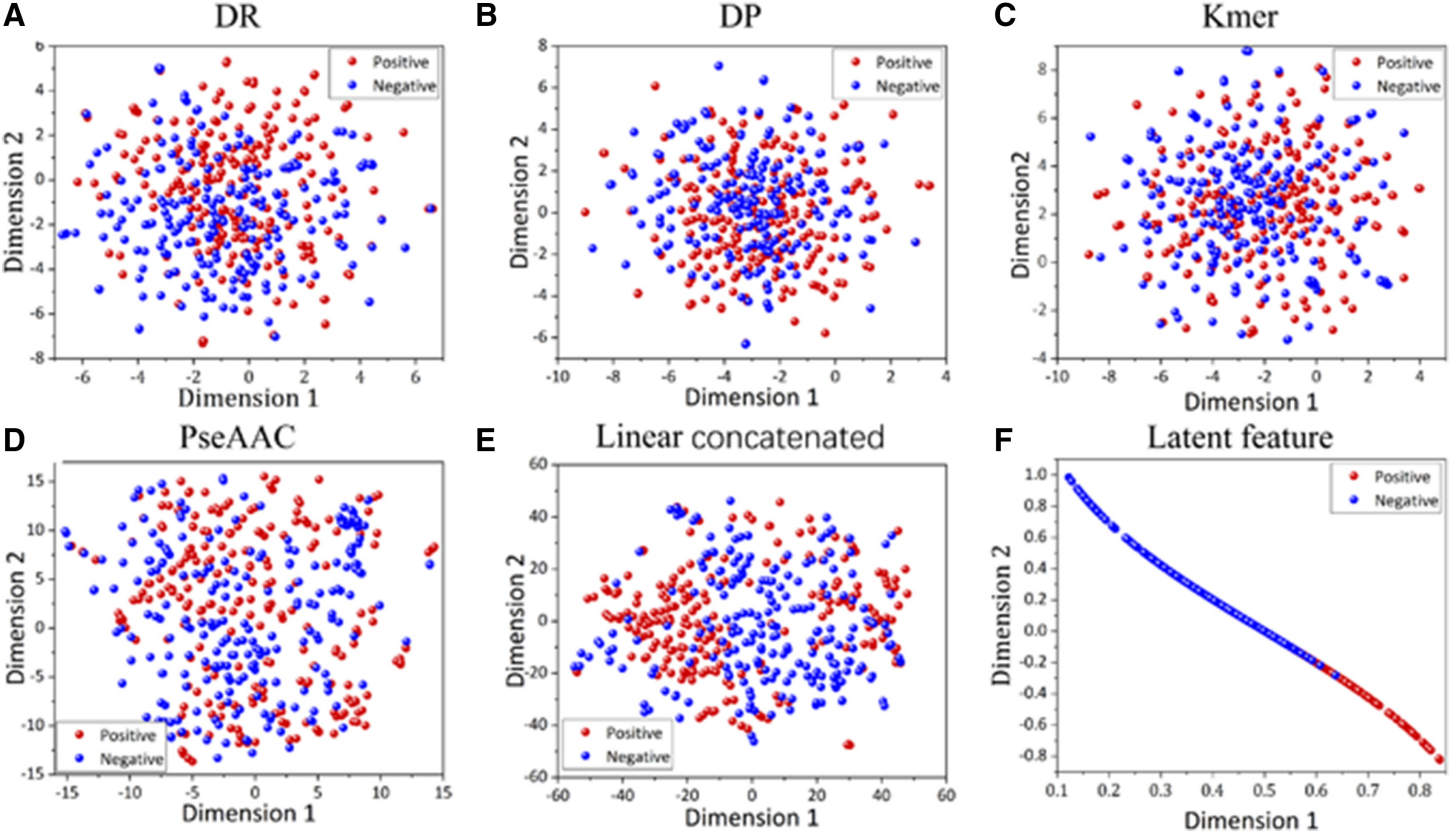
Feature visualization of ToxPre-2L and other four manual features. (A–F) Feature visualization of DR, DP, Kmer, Pse-AAC, linear concatenated feature, and latent features, respectively.

In this section, we perform the interpretability analysis of ToxPre-2L by visualizing the feature representation ability. We visualized all four features, the linearly concatenated features obtained by fuzing the four individual features, and the latent feature based on the proposed method. The construction of the latent feature subspace, which is shared with the multi-view features, is achieved through the employment of the MVTL algorithm. Subsequently, t-SNE (Van der Maaten and Hinton 2008) is applied to reduce the dimensions of diverse view features to two dimensions, facilitating the visualization of their distribution. The results, as depicted in Fig. 4, reveal the distinguishable information of latent features between TXP sequences and Non-TXP sequences. This observation suggests that the latent features automatically generated by ToxPre-2L possess discriminative properties conducive to peptide toxicity prediction.

### 3.5 Sensitivity to hyper-parameters

**Figure 5.**
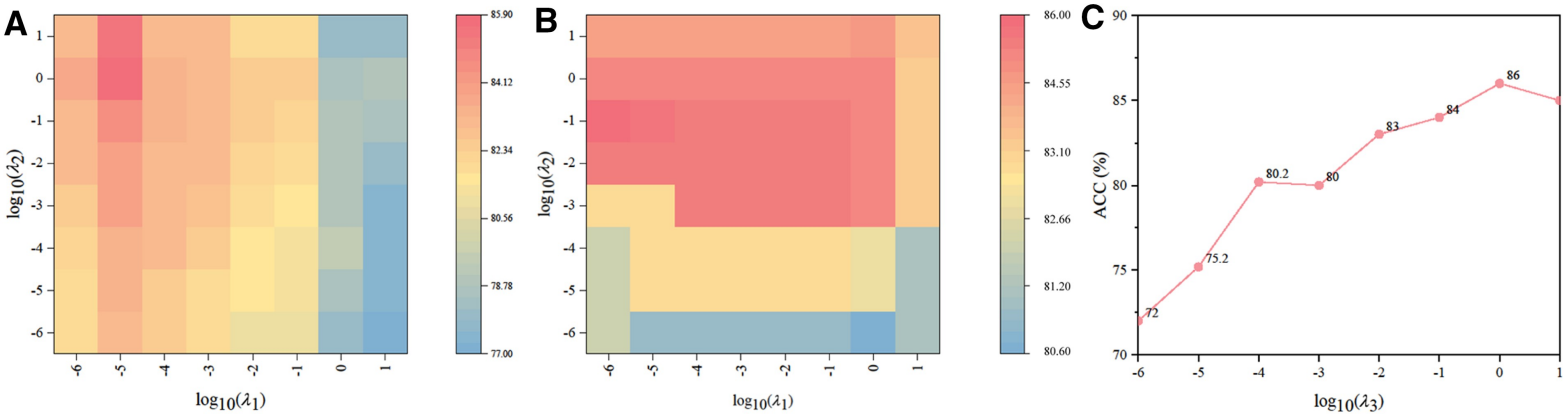
Sensitivity analysis to the hyper-parameters $\lambda_{1},\lambda_{2}$, and $\lambda_{3}$ of the ToxPre-2L on the datasets S and $S^{\mathrm{TXP}}$. (A) ACC values of the AdaptMVTL model with different combinations $\lambda_{1}$ and $\lambda_{2}$ on the dataset S. (B) ACC values of MLMVTLowRankBin model with different combinations $\lambda_{1}$ and $\lambda_{2}$ by fixing $\lambda_{3}$ on the dataset $S^{\mathrm{TXP}}$. (C) ACC values of MLMVTLowRankBin model with different $\lambda_{3}$ by fixing $\lambda_{1}$ and $\lambda_{2}$ on the dataset $S^{\mathrm{TXP}}$.

In this section, we evaluated the first-level stage subpredictor AdaptMVTL of ToxPre-2L and the second-level stage subpredictor MLMVTLowRankBin of ToxPre-2L to conduct sensitivity analysis to the hyperparameters $\left(\lambda_{1},\lambda_{2}\right)$ and $\left(\lambda_{1},\lambda_{2},\lambda_{3}\right)$, respectively. The parameters $\lambda_{1},\lambda_{2}$, and $\lambda_{3}$ are in the range of $\left\{10^{-6},10^{-5},10^{-4},10^{-3},10^{-2},10^{-1},1,10^{1}\right\}$. We adopted the grid search strategy to optimize the regularization parameters through a 10-fold cross-validation strategy on the datasets S and $S^{\mathrm{TXP}}$. The parameters of the proposed method were optimized on the validation set, which is independent with the training set and test set. Because of page limitation, Fig. 5 shows the ACC values for our model across different combinations of $\lambda_{1},\lambda_{2}$, and $\lambda_{3}$. From Fig. 5A and B, we selected two candidate sets $\left\{10^{-6},10^{-5}\right\}$ and $\left\{10^{-1},1,10^{1}\right\}$ for parameters $\lambda_{1}$ and $\lambda_{2}$, respectively. We perform the proposed method with various values of the two parameters, $\lambda_{1}$ and $\lambda_{2}$ in order to identify the best combination of these parameters in the 2D space formed by their candidate values. After determining the optimal combination of $\lambda_{1}$ and $\lambda_{2}$, we can fix these values and test different values of $\lambda_{3}$ to find its optimal value. As shown in Fig. 5C, the proposed method exhibits a relatively low sensitivity to the selection of the parameter $\lambda_{3}$ within the range $\left[10^{-1},10^{1}\right]$ to some extent. This process allows us to achieve the best combination of all three parameters. Finally, we conduct experiments using the selected parameters and present the results for comparison.

### 3.6 Complexity and computational time cost

The computational complexity of ToxPre-2L is analyzed as follows: for the first-level subpredictor AdaptMVTL [Equation (5)], the dominant operations involve updating $P^{\left(d\right)}$ with $Ο(Dn^{2}s)$ complexity and tensor z through 3D fast Fourier transformation (FFT)/inverse FFT computations requiring $Ο(Dn^{2}\log\left(n\right)+D^{2}n^{2})$ operations, while subsequent basic matrix operations are negligible, yielding an overall complexity of $Ο(\tau Dn^{2}(D+\log\left(n\right)+s))$ where τ represents iteration count. For the second-level subpredictor MLMVTLowRankBin [Equation (9)], each iteration’s computational cost is primarily determined by the gradient function update $\nabla f(P^{\left(d\right)})$ with $Ο(D(ns^{2}+s^{2}l+\mathrm{nsl}))$ and SVD decomposition of $P^{\left(d\right)}$ which, under typical multi-label classification conditions (s≫l), reduces to $Ο(Dsl^{2})$, along with an $Ο(Dn^{2}\log\left(n\right)+D^{2}n^{2})$ cost for updating tensor z, resulting in a total complexity of $Ο(\tau D(ns^{2}+s^{2}l+\mathrm{nsl}+sl^{2}+n^{2}\log\left(n\right)+Dn^{2}))$, with other basic operations contributing insignificantly to the overall computational burden.

We compared the computational time cost for all the comparing methods on the benchmark dataset S and $S^{\mathrm{TXP}}$. Tables 2 and 3 present the average computation times of different methods, clearly showing that our approach achieves the fastest processing speed among all compared methods on the two benchmark datasets. Specifically, on dataset S, our method completes the task in just 1.1 s, outperforming the second-best method ToxIBTL by 0.36 s. The ToxDL’s computational time exceeds 72 s, further highlighting the superior efficiency of our proposed method.

**Table 2.** The computational time (in seconds) of different methods on the benchmark dataset S.

Method	ToxinPred2	ToxDL	ToxIBTL	ToxPre-2L
Time	11.76	72.10	1.46	1.15

**Table 3.** The computational times (in seconds) of various methods on the benchmark dataset $S^{\mathrm{TXP}}$.

Method	Time
MIML-KNN	0.10
MIML-RBF	0.01
ML-KNN	0.08
RankSVM	0.02
LIFT	0.08
ETFC	2.68
IMFP	9.32
TPpred-LE	6.84
TPpred-SC	19.76
ToxPre-2L	0.02

## 4 Conclusion

We present a novel two-level predictor, ToxPre-2L, for predicting the function of TXP. To our knowledge, ToxPre-2L is the first computation predictor that adopts MVTL to predict TXPs and their multi-functional types. Its first level is to predict whether a query peptide is of TXP or not. If the output is yes, then the second level will predict its functional types. The proposed method utilized the MVTL and BR with robust low-rank constraint learning, which utilized the essential semantic information among different views and the label correlations between different multi-label information. The objective performance shows that ToxPre-2L outperforms the existing TXP prediction methods and several widely used multi-label predictors almost in all metrics. The experiential results confirmed that the proposed method has better performance for predicting toxicity peptides and their subfunction types. ToxPre-2L is a distinctive *in silico* method to predict the toxicity of peptides, offering valuable predictive insights. We anticipate that the advancement of ToxPre-2L will catalyze peptide-based drug discovery efforts.

## Supplementary Material

btaf489_Supplementary_Data

## Data Availability

The data is available at http://bliulab.net/ToxPre-2L.
