# Supplementary material for: Accurate prediction of toxicity peptide and its function using multi-view tensor learning and latent semantic learning framework
Source: Bioinformatics. 2025 Sep 4;41(9):btaf489. doi: 10.1093/bioinformatics/btaf489 (PMC12457739; doi:10.1093/bioinformatics/btaf489)
Supplement: btaf489_Supplementary_Data [file btaf489_supplementary_data.zip › Supplementary Material S1.docx]

**Supplementary Material S1**

**Details of feature extraction algorithms**

A peptide sequence $R$ can be expressed as

|  | $R=R_{1}R_{2}\cdots R_{L}$ | (1) |
| --- | --- | --- |

where $R_{i}$ is the $i$-th residue of short-long peptide $R$. $L$ is the length of the peptide $R$. In this study, four features were employed to represent the peptide, including the *K*-mer, distance-based residue (DR), distance pair (DP) and pseudo amino acid composition (Pse-AAC). The four features are introduced in detail as follows:

1. *K*-mer

*K*-mer feature is a widely used feature encode method in therapeutic peptides [1], protein fold recognition [2], etc. *K*-mer is the occurrence frequency of the substring with the length of the *K* along the peptide. For the substring, the *K*-mer is represented as

|  | $k$-mer=${N_{substring}}/L$ | (2) |
| --- | --- | --- |

where $N_{substring}$ is the occurrence number of substring appeared in the peptide $R$. In this study, the parameter *K* is 2 and the dimension of the *K*-mer is 400-D.

1. DR

DR feature utilizes the amino acid pairs distribution information with the distance spaces in the peptides [3]. DR calculates the frequency of amino acid pairs with the distance *LG* in the peptide. For example, in the peptide “TAI****M”, the amino acid pair “I****M” is a 4-spaced amino acid pair. In this study, the dimension of DR is 1220-D.

1. DP

DP feature captures the pseudo amino acid pairs occurrence frequency in the sequence [4]. DP can be represented as

|  | DP=*f*$\left( R_{i},R_{j}\left\lfloor d \right. \right)$ | (3) |
| --- | --- | --- |

where $R_{i}$ and $R_{j}$ are the $i$-th and $j$-th residues, respectively. $d$ represents the distance between $R_{i}$ and $R_{j}$ along the peptide. In this study, the dimension of DP is 602-D.

1. PseAAC

PseAAC utilizes the physicochemical information and sequence information to represent the peptide sequence [5]. The PseAAC can be defined as

|  | $\mathrm{PseAAC}=\left[ S_{1},\cdots,S_{\mu},\cdots S_{20+2\lambda} \right]$ | (4) |
| --- | --- | --- |

where $S_{\mu}$ is calculated as

|  | $S_{\mu}=\left\{ \begin{matrix} \frac{f_{i}}{\sum_{i=1}^{20} f_{i}+\omega\sum_{j=1}^{\lambda} \gamma_{j}},\mu\in\left[ 1,20 \right] \\ \frac{\omega\gamma_{\mu-20}}{\sum_{i=1}^{20} f_{i}+\omega\sum_{j=1}^{\lambda} \gamma_{j}},\mu\in\left[ 21,20+2\lambda\right] \end{matrix} \right.$ | (5) |
| --- | --- | --- |

where $f_{i}$ represents the frequency of the amino acid $R_{i}$, $\gamma_{j}$ represents the physicochemical information, including hydrophobicity and hydrophilicity information. In this study, the parameter $\lambda$ is 3 and the dimension of PseAAC is 26-D.


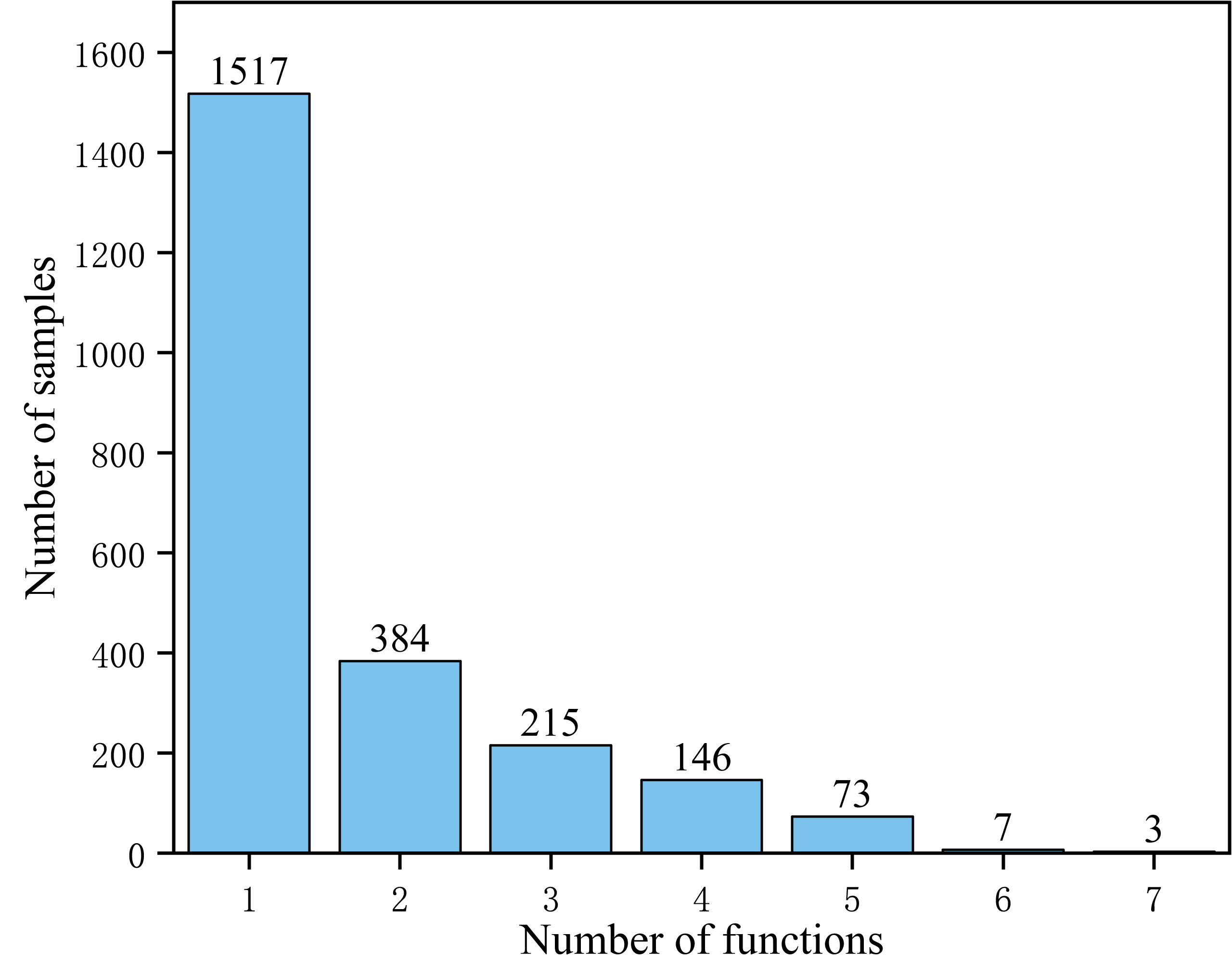


**Fig.S1**. The number of functions assigned to each sequence in the second multi-functional dataset $\mathbb{S}^{\mathrm{TXP}}$





**Fig. S2**. The prediction results of four predictors were based on the 10-fold cross-validation strategy in terms of SN, SP, MCC and F1 metrics. The different methods are used to identify TXP and Non-TXP.

**Table S1.** The hyper-parameter sets of the existing predictors in identifying TXP and Non-TXP

| **Method** | **Parameters** |
| --- | --- |
| ToxinPred2 | class_weight='balanced', criterion='entropy',  max_depth=50, max_features='log2',  n_estimators=1000, random_state=42,  motif analysis: default parameters, threshold=0.6 |
| ToxDL | learning_rate=0.001, epoch=10, dropout=0.5,  conv_filters=200, pool_size=3, pool_stride=3,  k_max_pooling=10, Optimizer: Adam |
| ToxIBTL | learning_rate=0.0001, epoch=500, dropout=0.5,  conv_filters=128, bigru_layers=2, bidirectional=True,  bigru_dropout=0.2, Optimizer: Adam |

**Table S2**. Hyper-parameter configurations of deep learning-based TXP multifunctional predictors

| **Method** | **Parameters** |
| --- | --- |
| iMFP-LG | pad_token_id = 0, batch_size = 32, max_sen_len = None,  num_labels = 21(MFTP)/5(MFBP), epochs = 100,  learning_rate = 0.00005(BERT)/0. 0.0005(GAT),  tokenizer=bert_tokenizer, max_position_embeddings=512,  Optimizer:Adam |
| TPpred-LE | Learning_rate=0.0001, epoch=30, dropout=0.1, max_lenth=50,  model_dim=256, n_class=15, nhead=4, n_enc_layers=2,  n_dec_layers=2, n_dec_layers=2, Dropout rate=0.1,  Optimizer:Adam |
| TPpred-SC | batch_size=128, epochs = 300, learning_rate=0.01,  lr_decay_rate=0.1, parameter for random mutation=0.1,  parameter for jaccard=0.1, temperature for loss function=0.07,  Optimizer:SGD |

**References**

[1] Y. Guo, K. Yan, H. Lv, and B. Liu, “PreTP-EL: prediction of therapeutic peptides based on ensemble learning,” *Briefings in Bioinformatics,* vol. 22, no. 6, pp. bbab358, Aug 28, 2021.

[2] K. Yan, X. Fang, Y. Xu, and B. Liu, “Protein Fold Recognition based on Multi-view Modeling,” *Bioinformatics,* vol. 35, no. 17, pp. 2982-2990, 2019.

[3] B. Liu, D. Zhang, R. Xu, J. Xu, X. Wang, Q. Chen, Q. Dong, and K.-C. Chou, “Combining evolutionary information extracted from frequency profiles with sequence-based kernels for protein remote homology detection,” *Bioinformatics,* vol. 30, no. 4, pp. 472-479, 2014.

[4] B. Liu, J. Xu, X. Lan, R. Xu, J. Zhou, X. Wang, and K.-C. Chou, “iDNA-Prot| dis: Identifying DNA-binding proteins by incorporating amino acid distance-pairs and reduced alphabet profile into the general pseudo amino acid composition,” *PloS one,* vol. 9, no. 9, pp. e106691, 2014.

[5] H. B. Shen, and K. C. Chou, “PseAAC: a flexible web server for generating various kinds of protein pseudo amino acid composition,” *Analytical biochemistry,* vol. 373, no. 2, pp. 386-388, Feb 15, 2008.
