# Supplementary material for: Accurate prediction of toxicity peptide and its function using multi-view tensor learning and latent semantic learning framework
Source: Bioinformatics. 2025 Sep 4;41(9):btaf489. doi: 10.1093/bioinformatics/btaf489 (PMC12457739; doi:10.1093/bioinformatics/btaf489)
Supplement: btaf489_Supplementary_Data [file btaf489_supplementary_data.zip › Supplementary Material S2.docx]

**Supplementary Material S2**

**Details of optimization algorithm of ToxPre-2L**

We introduce the optimization algorithm for ToxPre-2L, employing two distinct optimization strategies to tackle AdaptMVTL and MLMVTLowRankBin, respectively.

1. Optimization of AdaptMVTL model

We utilized the auxiliary variable $\boldsymbol{Z}^{\left( d \right)}$ as a surrogate of $\mathbf{X}_{\mathrm{tr}}^{\left( d \right)}\mathbf{P}^{\left( d \right)}$ in the tensor nuclear norm of $\mathcal{z}$. The formular of AdaptMVTL can be solved as

| $\min_{a^{\left( d \right)}, \mathbf{P}^{\left( d \right)},\mathcal{z}} \sum_{d=1}^{D} {a^{\left( d \right)}\left\Vert\mathbf{X}_{\mathrm{tr}}^{\left( d \right)}\mathbf{P}^{\left( d \right)}-\mathbf{Y} \right\Vert}_{F}^{2}+\lambda_{1}\sum_{d=1}^{D} \left\Vert\mathbf{P}^{\left( d \right)} \right\Vert_{2}^{2}+\lambda_{2}\left\Vert\mathcal{z} \right\Vert_{⊛}+\gamma\left\Vert a \right\Vert_{2}^{2}$  $s.t.a^{\left( d \right)}\geq0, \boldsymbol{Z}^{\left( d \right)}=\mathbf{X}_{\mathrm{tr}}^{\left( d \right)}\mathbf{P}^{\left( d \right)}, \sum_{d=1}^{D} a^{\left( d \right)}=1$ | (1) |
| --- | --- |

where the tesnsor variable $\mathcal{z}$ is constructed by $\left\{ \boldsymbol{Z}^{\left( d \right)} \right\}_{d=1}^{D}$. Eq.(9) can be transformed as the augmented Lagrangian formula

| $\min_{a^{\left( d \right)}, \mathbf{P}^{\left( d \right)},\mathcal{z}}\sum_{d=1}^{D} {a^{\left( d \right)}\left\Vert\mathbf{X}_{\mathrm{tr}}^{\left( d \right)}\mathbf{P}^{\left( d \right)}-\mathbf{Y} \right\Vert}_{F}^{2} +\lambda_{1}\sum_{d=1}^{D} \left\Vert\mathbf{P}^{\left( d \right)} \right\Vert_{2}^{2}+\lambda_{2}\left\Vert\mathcal{z} \right\Vert_{⊛}+\frac{\mu}{2}\sum_{d=1}^{D} \left\Vert\mathbf{X}_{\mathrm{tr}}^{\left( d \right)}\mathbf{P}^{\left( d \right)}\boldsymbol{-}\boldsymbol{Z}^{\left( d \right)}+\frac{\mathbf{M}^{\left( d \right)}}{\mu} \right\Vert_{F}^{2}+\gamma\left\Vert a \right\Vert_{2}^{2}$  $s.t. a^{\left( d \right)}\geq0,\sum_{d=1}^{D} a^{\left( d \right)}=1$ | (2) |
| --- | --- |

where $\mathbf{M}^{\left( d \right)}$ is the Lagrangian multiplier and $\mu$ is a penalty parameter. Following this, optimization of the variables is performed utilizing the specified update rule.

**Step 1: Updating** $\mathbf{P}^{\left( d \right)}$: Assuming that $D$ view features are independent, we considered the $d$-th view training dataset feature. Fix the other variables and update $\mathbf{P}^{\left( d \right)}$ by solving the following problem

| $\mathbf{P}^{\left( d \right)+}\boldsymbol{=}\underset{\mathbf{P}^{\left( d \right)}}{\mathrm{argmin}} {a^{\left( d \right)}\left\Vert\mathbf{X}_{\mathrm{tr}}^{\left( d \right)}\mathbf{P}^{\left( d \right)}-\mathbf{Y} \right\Vert}_{F}^{2}+\lambda_{1}\left\Vert\mathbf{P}^{\left( d \right)} \right\Vert_{2}^{2}+\frac{\mu}{2}\left\Vert\mathbf{X}_{\mathrm{tr}}^{\left( d \right)}\mathbf{P}^{\left( d \right)}\boldsymbol{-}\boldsymbol{Z}^{\left( d \right)}+\frac{\mathbf{M}^{\left( d \right)}}{\mu} \right\Vert_{F}^{2}$ | (3) |
| --- | --- |

where the rest terms irrelated with the $\mathbf{P}^{\left( d \right)}$ are considered as the constants. The problem Eq.(3) is a regularized least squared problem and the solution turns out to be

| $\mathbf{P}^{\left( d \right)+}\boldsymbol{=}\left( 2a^{\left( d \right)}\mathbf{X}_{\mathrm{tr}}^{\left( d \right)}\mathbf{Y}+\mu\mathbf{X}_{\mathrm{tr}}^{\left( d \right)T}\left( \boldsymbol{Z}^{\left( d \right)}-\frac{\mathbf{M}^{\left( d \right)}}{\mu} \right) \right) \left( \left( 2a^{\left( d \right)}+\mu\right)\mathbf{X}_{\mathrm{tr}}^{\left( d \right)T}\mathbf{X}_{\mathrm{tr}}^{\left( d \right)}\boldsymbol{+}2\lambda_{1}I \right)^{-1}$ | (4) |
| --- | --- |

**Step 2: Updating** $\mathcal{z}$*:* According to the problem Eq.(2), fix the other variables and the *tensor variable* $\mathcal{z}$ is solved by the following problem

| $\mathcal{z}^{+}\boldsymbol{=}\underset{\mathcal{z}}{\mathrm{argmin}} \lambda_{2}\left\Vert\mathcal{z} \right\Vert_{⊛}+\frac{\mu}{2}\sum_{d=1}^{D} \left\Vert\mathbf{X}_{\mathrm{tr}}^{\left( d \right)}\mathbf{P}^{\left( d \right)}\boldsymbol{-}\boldsymbol{Z}^{\left( d \right)}+\frac{\mathbf{M}^{\left( d \right)}}{\mu} \right\Vert_{F}^{2}$ | (5) |
| --- | --- |

Defined $\mathbf{A}^{\left( d \right)}\mathbf{=X}_{\mathrm{tr}}^{\left( d \right)}\mathbf{P}^{\left( d \right)}+\frac{\mathbf{M}^{\left( d \right)}}{\mu}$ and the tensor $\mathcal{A=}\left\{ \mathbf{A}^{\left( d \right)} \right\}_{d=1}^{D}$, then the problem Eq.(5) can be transformed into

| $\mathcal{z}^{+}\boldsymbol{=}\underset{\mathcal{z}}{\mathrm{argmin}} \left\Vert\mathcal{z} \right\Vert_{⊛}+\frac{\mu}{2\lambda_{2}}\left\Vert\mathcal{z}\mathcal{-A} \right\Vert_{F}^{2}$ | (6) |
| --- | --- |

According to the [1], the tensor variable $\mathcal{z}$ has the closed-form solution:

| $\mathcal{z}^{+}\boldsymbol{=}\mathcal{u\times}\Upsilon_{\frac{m\times\lambda_{2}}{\mu}}(S)\times v^{T}$ | (7) |
| --- | --- |

where $\Upsilon_{\frac{m\times\lambda_{2}}{\mu}}(S)=S\mathcal{\times I}$, $\mathcal{I}$is a f-diagonal tensor and its diagonal element is denoted by $\mathcal{I}_{f}\left( i,i,i \right)=\left( 1-\left( \frac{m\lambda_{2}}{\mu S_{f}^{\left( j \right)}\left( i,i \right)} \right) \right)$ in the Fourier domain [1].

**Step 3: Updating** $a^{\left( d \right)}$: The variable $a^{\left( d \right)}$ corresponding to the $d$*-*th view is transformed into:

| $a^{\left( d \right)+}\boldsymbol{=}\underset{a^{\left( d \right)}}{\mathrm{argmin}} \sum_{d=1}^{D} a^{\left( d \right)}W^{\left( d \right)}+\gamma\left\Vert a \right\Vert_{2}^{2} s.t. a^{\left( d \right)}\geq0,\sum_{d=1}^{D} a^{\left( d \right)}=1$ | (8) |
| --- | --- |

where $W^{\left( d \right)}=\left\| \mathbf{X}_{\mathrm{tr}}^{\left( d \right)}\mathbf{P}^{\left( d \right)}-\mathbf{Y} \right\|_{F}^{2}$, the portion $\gamma\left\| a \right\|_{2}^{2}$ is used to smooth the weight distribution and avoid the futile solution [2]. Then the problem Eq.(8) is rewritten as follows:

| $a^{\left( d \right)+}\boldsymbol{=}\underset{a^{\left( d \right)}}{\mathrm{argmin}} \left\Vert a+\frac{W}{2\gamma} \right\Vert_{2}^{2} s.t. a^{\left( d \right)}\geq0,\sum_{d=1}^{D} a^{\left( d \right)}=1$ | (9) |
| --- | --- |

Then we utilized the quadprog program to solve the off-the-shelf quadratic programming.

**Step 4: Updating Lagrangian multiplier** $\mathbf{M}^{\left( d \right)}$ **and penalty parameter** $\mu$: The $\mathbf{M}^{\left( d \right)}$ and $\mu$ can be updated as follows:

| $\mathbf{M}^{\left( d \right)\boldsymbol{+}}\boldsymbol{=}\mathbf{M}^{\left( d \right)}\boldsymbol{+}\mu\left( \mathbf{X}_{\mathrm{tr}}^{\left( d \right)}\mathbf{P}^{\left( d \right)}\boldsymbol{-}\boldsymbol{Z}^{\left( d \right)} \right)$ | (10) |
| --- | --- |
| $\mu\boldsymbol{=}\min\left( \rho\mu,\mu_{\max} \right)$ | (11) |

where $\rho$ and $\mu_{\max}$ are constants.

Furthermore, Algorithm 1 provides the summary of AdaptMVTL, offering its methodology for addressing Eq.(1).

| Algorithm 1: Optimization of AdaptMVTL for the first-level stage sub-predictor of ToxPre-2L |
| --- |
| **Input:** $\left\{ \mathbf{X}_{\mathrm{tr}}^{\left( 1 \right)},\cdots,\mathbf{X}_{\mathrm{tr}}^{\left( D \right)} \right\}$ with$D$ view feature matrices and the label set $\mathbf{Y}$. A test sample of $D$ views $\left\{ x_{\mathrm{tt}}^{\left( d \right)} \right\}_{d=1}^{D}$. Parameters $\lambda_{1}, \lambda_{2}$.  1: Initialization: $\mathbf{P}^{\left( d \right)}$is initialized by the conventional linear regression model. $\mathbf{M}^{\left( d \right)}$ is the zero matrix, $\mathbf{Z}^{\left( d \right)}=\mathbf{X}_{\mathrm{tr}}^{\left( d \right)}\mathbf{P}^{\left( d \right)},a^{\left( d \right)}=\frac{1}{D}{,{,\mu}_{1}=0.4, \mu}_{\max}={10}^{6},\rho=1.1,\gamma=500$. |
| 2: While not converged do |
| 3: While $d\leq D$ do |
| 3: Update $\mathbf{P}^{\left( d \right)}$ by solving Eq. (4); |
| 4: Update $\mathbf{M}^{\left( d \right)}$ by solving Eq. (10); |
| 5: Update $a^{\left( d \right)}$ by solving the quadratic program; |
| 6: End while |
| 7: Update $\mathcal{z}$ by solving Eq. (7); |
| 8: Update $\mu$ by solving Eq. (11); |
| 9: End while |
| **Output:** Prediction the binary labels (TXP and Non-TXP) of test peptide sequence. |

### 2. Optimization of MLMVTLowRankBin model

In this section, we also utilized the auxiliary variable $\boldsymbol{Z}^{\left( d \right)}$ as a surrogate of $\mathbf{X}_{\mathrm{tr}}^{\left( d \right)}\mathbf{P}^{\left( d \right)}$ within the tensor nuclear norm of $\mathcal{z}$. The MLMVTLowRankBin model can be solved as

| $\begin{aligned} \min_{a^{\left( d \right)}, \mathbf{P}^{\left( d \right)},\mathcal{z}}\sum_{d=1}^{D} {a^{\left( d \right)}\left\Vert\mathbf{X}_{\mathrm{tr}}^{\left( d \right)}\mathbf{P}^{\left( d \right)}-\mathbf{Y} \right\Vert}_{F}^{2}+\sum_{d=1}^{D} \frac{1}{2}\left\Vert\left( \left\vert\mathbf{E}-\mathbf{Y⨀}\left( \mathbf{X}_{\mathrm{tr}}^{\left( d \right)}\mathbf{P}^{\left( d \right)} \right) \right\vert_{\boldsymbol{+}} \right)^{\boldsymbol{2}} \right\Vert_{1}+ \\ \lambda_{1}\sum_{d=1}^{D} \left\Vert\mathbf{P}^{\left( d \right)} \right\Vert_{2}^{2}+\lambda_{2}\left\Vert\mathcal{z} \right\Vert_{⊛}+\lambda_{3}\sum_{d=1}^{D} \left\Vert\mathbf{P}^{\left( d \right)} \right\Vert_{*}+\gamma\left\Vert a \right\Vert_{2}^{2} \\ s.t. a^{\left( d \right)}\geq0,\sum_{d=1}^{D} a^{\left( d \right)}=1,\boldsymbol{Z}^{\left( d \right)}=\mathbf{X}_{\mathrm{tr}}^{\left( d \right)}\mathbf{P}^{\left( d \right)} \\ \end{aligned}$ | (12) |
| --- | --- |

Similar to the previous strategy, Eq.(12) can be transformed into

| $\begin{aligned} \min_{a^{\left( d \right)}, \mathbf{P}^{\left( d \right)},\mathcal{z}}\sum_{d=1}^{D} {a^{\left( d \right)}\left\Vert\mathbf{X}_{\mathrm{tr}}^{\left( d \right)}\mathbf{P}^{\left( d \right)}-\mathbf{Y} \right\Vert}_{F}^{2}+\sum_{d=1}^{D} \frac{1}{2}\left\Vert\left( \left\vert\mathbf{E}-\mathbf{Y⨀}\left( \mathbf{X}_{\mathrm{tr}}^{\left( d \right)}\mathbf{P}^{\left( d \right)} \right) \right\vert_{\boldsymbol{+}} \right)^{\boldsymbol{2}} \right\Vert_{1}+ \\ \lambda_{1}\sum_{d=1}^{D} \left\Vert\mathbf{P}^{\left( d \right)} \right\Vert_{2}^{2}+\lambda_{2}\left\Vert\mathcal{z} \right\Vert_{⊛}+\lambda_{3}\sum_{d=1}^{D} \left\Vert\mathbf{P}^{\left( d \right)} \right\Vert_{*}+\gamma\left\Vert a \right\Vert_{2}^{2} \\ +\frac{\mu}{2}\sum_{d=1}^{D} \left\Vert\mathbf{X}_{\mathrm{tr}}^{\left( d \right)}\mathbf{P}^{\left( d \right)}\boldsymbol{-}\boldsymbol{Z}^{\left( d \right)}+\frac{\mathbf{M}^{\left( d \right)}}{\mu} \right\Vert_{F}^{2} s.t. a^{\left( d \right)}\geq0,\sum_{d=1}^{D} a^{\left( d \right)}=1 \\ \end{aligned}$ | (13) |
| --- | --- |

It is readily apparent that the optimization process for MLMVTLowRankBin closely parallels that of AdaptMVTL, except for the $\mathbf{P}^{\left( d \right)}$. Subsequently, while fixing all other variables, the update of $\mathbf{P}^{\left( d \right)}$ is undertaken through the resolution of the ensuing problem:

| $\begin{aligned} \min_{\mathbf{P}^{\left( d \right)}}\sum_{d=1}^{D} {a^{\left( d \right)}\left\Vert\mathbf{X}_{\mathrm{tr}}^{\left( d \right)}\mathbf{P}^{\left( d \right)}-\mathbf{Y} \right\Vert}_{F}^{2}+\sum_{d=1}^{D} \frac{1}{2}\left\Vert\left( \left\vert\mathbf{E}-\mathbf{Y⨀}\left( \mathbf{X}_{\mathrm{tr}}^{\left( d \right)}\mathbf{P}^{\left( d \right)} \right) \right\vert_{\boldsymbol{+}} \right)^{\boldsymbol{2}} \right\Vert_{1} \\ +\lambda_{1}\sum_{d=1}^{D} \left\Vert\mathbf{P}^{\left( d \right)} \right\Vert_{2}^{2}+\lambda_{3}\sum_{d=1}^{D} \left\Vert\mathbf{P}^{\left( d \right)} \right\Vert_{*} \\ +\frac{\mu}{2}\sum_{d=1}^{D} \left\Vert\mathbf{X}_{\mathrm{tr}}^{\left( d \right)}\mathbf{P}^{\left( d \right)}\boldsymbol{-}\boldsymbol{Z}^{\left( d \right)}+\frac{\mathbf{M}^{\left( d \right)}}{\mu} \right\Vert_{F}^{2} \\ \end{aligned}$ | (14) |
| --- | --- |

The problem Eq.(14) is convex but non-smooth due to the low-rank norm. We utilized the accelerated proximal gradient (APG) strategy [3] to optimize the problem. Specifically, we utilized a generalized APG framework to address the non-smooth convex problem

| $\min_{\mathbf{P}^{\left( d \right)}\boldsymbol{\in}\mathcal{H}} \mathcal{F}\left( \mathbf{P}^{\left( d \right)} \right)=f\left( \mathbf{P}^{\left( d \right)} \right)+g\left( \mathbf{P}^{\left( d \right)} \right)$ | (15) |
| --- | --- |

where $f\left( \mathbf{P}^{\left( d \right)} \right): \mathcal{H}\mathbb{\to R}$ is a convex and smooth function and $g\left( \mathbf{P}^{\left( d \right)} \right)\mathcal{:H}\mathbb{\to R}$ represents the convex and non-smooth function. The gradient function $\nabla f\left( \right)$ is Lipschitz continuous, i.e. $\forall\mathbf{P}_{\boldsymbol{1}}^{\left( d \right)}\boldsymbol{,}\mathbf{P}_{\boldsymbol{2}}^{\left( d \right)}\boldsymbol{\in}\mathcal{H}$, $\left\| \nabla f\left( \mathbf{P}_{\boldsymbol{1}}^{\left( d \right)} \right)-\nabla f\left( \mathbf{P}_{\boldsymbol{2}}^{\left( d \right)} \right) \right\|\boldsymbol{\leq}L_{f}\left\| \triangle\mathbf{P}^{\left( d \right)} \right\|$, where $\triangle\mathbf{P}^{\left( d \right)}\boldsymbol{=}\mathbf{P}_{\boldsymbol{1}}^{\left( d \right)}\boldsymbol{-}\mathbf{P}_{\boldsymbol{2}}^{\left( d \right)}$and $L_{f}$ denotes the Lipschitz constant. Then the problem Eq.(15) can be divided into as follows,

| $f\left( \mathbf{P}^{\left( d \right)} \right)=\sum_{d=1}^{D} {a^{\left( d \right)}\left\Vert\mathbf{X}_{\mathrm{tr}}^{\left( d \right)}\mathbf{P}^{\left( d \right)}-\mathbf{Y} \right\Vert}_{F}^{2}+\lambda_{1}\sum_{d=1}^{D} \left\Vert\mathbf{P}^{\left( d \right)} \right\Vert_{2}^{2}+\sum_{d=1}^{D} \frac{1}{2}\left\Vert\left( \left\vert\mathbf{E}-\mathbf{Y⨀}\left( \mathbf{X}_{\mathrm{tr}}^{\left( d \right)}\mathbf{P}^{\left( d \right)} \right) \right\vert_{\boldsymbol{+}} \right)^{\boldsymbol{2}} \right\Vert_{1}+\frac{\mu}{2}\sum_{d=1}^{D} \left\Vert\mathbf{X}_{\mathrm{tr}}^{\left( d \right)}\mathbf{P}^{\left( d \right)}\boldsymbol{-}\boldsymbol{Z}^{\left( d \right)}+\frac{\mathbf{M}^{\left( d \right)}}{\mu} \right\Vert_{F}^{2}$  $g\left( \mathbf{P}^{\left( d \right)} \right)=\lambda_{3}\sum_{d=1}^{D} \left\Vert\mathbf{P}^{\left( d \right)} \right\Vert_{*}$ | (16) |
| --- | --- |

Assuming that the information of $D$ views is irrelevant to each other, we considered the $d$-th view training peptide sequences. Then we computed the gradient function of $f\left( \mathbf{P}^{\left( d \right)} \right)$ and then calculated the corresponding Lipschitz constant. Firstly, we obtained the gradient function $\nabla f\left( \mathbf{P}^{\left( d \right)} \right)$ as follows,

| $\nabla f\left( \mathbf{P}^{\left( d \right)} \right)={2a}^{\left( d \right)}\mathbf{X}_{\mathrm{tr}}^{\left( d \right)T}\left( \mathbf{X}_{\mathrm{tr}}^{\left( d \right)}\mathbf{P}^{\left( d \right)}-\mathbf{Y} \right)+2\lambda_{1}\mathbf{P}^{\left( d \right)}+\mathbf{X}_{\mathrm{tr}}^{\left( d \right)T}\left( \left\vert\mathbf{E}-\mathbf{Y⨀}\left( \mathbf{X}_{\mathrm{tr}}^{\left( d \right)}\mathbf{P}^{\left( d \right)} \right) \right\vert_{\boldsymbol{+}}\mathbf{⨀}\left( \boldsymbol{-}\mathbf{Y} \right) \right)\boldsymbol{+}\mu\mathbf{X}_{\mathrm{tr}}^{\left( d \right)T}\left( \mathbf{X}_{\mathrm{tr}}^{\left( d \right)}\mathbf{P}^{\left( d \right)}\boldsymbol{-}\boldsymbol{Z}^{\left( d \right)}+\frac{\mathbf{M}^{\left( d \right)}}{\mu} \right)$ | (17) |
| --- | --- |

**Theorem 1** The Lipschitz constant of $\nabla f\left( \mathbf{P}^{\left( d \right)} \right)$ with respect to $\mathbf{P}^{\left( d \right)}$ in Eq.(17) is

| $L_{f}=\sqrt{2\left\Vert\left( {2a}^{\left( d \right)}+\mu\right)\mathbf{X}_{\mathrm{tr}}^{\left( d \right)T}\mathbf{X}_{\mathrm{tr}}^{\left( d \right)}+2\lambda_{1}\mathbf{I} \right\Vert_{F}^{2}+2\left( \left\Vert\mathbf{X}_{\mathrm{tr}}^{\left( d \right)} \right\Vert_{F}^{2} \right)^{2}}$ | (18) |
| --- | --- |

Detailed proof process to **Theorem 1** can be found in the Appendix Note1. Furthermore, we utilized the singular value thresholding (SVT) operator strategy [4] to optimize the $g\left( \mathbf{P}^{\left( d \right)} \right)$ function.

| $\mathbf{prog}_{\mathcal{l}}\left( \mathbf{P}^{\left( d \right)} \right)\boldsymbol{=}\mathbf{U}\boldsymbol{\Sigma}_{\mathcal{l}}\mathbf{V}^{T}$ | (19) |
| --- | --- |

where $\mathbf{U}$ and $\mathbf{V}$ are unitary matrices based on the singular value decomposition of $\mathbf{P}^{\left( d \right)}$, $\boldsymbol{\Sigma}_{\mathcal{l}}$ is a diagonal matrix with $\left( \boldsymbol{\Sigma}_{\mathcal{l}} \right)_{ii}\boldsymbol{=}max(0,\boldsymbol{\Sigma}_{ii}-\mathcal{l})$.

The optimization $\mathbf{P}^{\left( d \right)}$ is outlined as follows.

| ${\mathbf{P}_{t}^{\left( d \right)}\mathbf{=prog}}_{\left( {\lambda_{3}}/{L_{f}} \right)}\left( \mathbf{G}_{t}\boldsymbol{-}\frac{1}{L_{f}}\nabla_{\mathbf{G}_{t}}f\left( \mathbf{G}_{t} \right) \right)$ | (20) |
| --- | --- |
| $b_{t+1}=\frac{1+\sqrt{1+4b_{t}^{2}}}{2}$ | (21) |
| $\mathbf{G}_{t+1}=\mathbf{P}_{t}^{\left( d \right)}\boldsymbol{+}\frac{b_{t}-1}{b_{t+1}}\left( \mathbf{P}_{t}^{\left( d \right)}\boldsymbol{-}\mathbf{P}_{t-1}^{\left( d \right)} \right)$ | (22) |

In addition, the optimization solution for $\mathcal{z}$, $\mathbf{M}^{\left( d \right)}\boldsymbol{,}$ $a^{\left( d \right)}$ and $\mu$ mirror those of AdaptMVTL. The comprehensive procedures for achieving the optimal solution of MLMVTLowRankBin are encapsulated in **Algorithm 2**.

| **Algorithm 2:** Optimization of MLMVTLowRankBin for the second-level stage sub-predictor of ToxPre-2L |
| --- |
| **Input:** $\left\{ \mathbf{X}_{\mathrm{tr}}^{\left( 1 \right)},\cdots,\mathbf{X}_{\mathrm{tr}}^{\left( D \right)} \right\}$ with$D$ view feature matrices and the label set $\mathbf{Y}$. A test sample of $D$ views $\left\{ x_{\mathrm{tt}}^{\left( d \right)} \right\}_{d=1}^{D}$. Parameters $\lambda_{1}, \lambda_{2},\lambda_{3}.$  1: Initialization: $\mathbf{P}^{\left( d \right)}$is initialized by the conventional linear regression model. $\mathbf{M}^{\left( d \right)}$and $\mathbf{G}_{\boldsymbol{1}}$are zero matrices$,\mathbf{Z}^{\left( d \right)}=\mathbf{X}_{\mathrm{tr}}^{\left( d \right)}\mathbf{P}^{\left( d \right)},a^{\left( d \right)}=\frac{1}{D}{{, \mu}_{1}=0.4,\mu}_{\max}={10}^{6},\rho=1.1,t=1,b_{1}=1,\gamma=500$. |
| 2: Calculate $L_{f}$ according to Eq.(18) |
| 3: While not converged do |
| 4: While $d\leq D$ do |
| 5: Calculate the gradient of $\nabla f\left( \mathbf{P}^{\left( d \right)} \right)$ via Eq.(17); |
| 6: Update $\mathbf{P}_{t}^{\left( d \right)}$ by solving Eq.(20); |
| 7: Update $b_{t+1}$ by solving Eq.(21) ; |
| 8: Update $\mathbf{G}_{t+1}$ by solving Eq.(22) ; |
| 9: Update $\mathbf{M}^{\left( d \right)}$ by solving Eq. (10); |
| 10: Update $a^{\left( d \right)}$ by solving the quadratic program; |
| 11: End while |
| 12: Update $\mathcal{z}$ by solving Eq. (7); |
| 13: Update $\mu$ by solving Eq. (11); |
| 14: $t=t+1$; |
| 15: End while |
| **Output:** Prediction the multi-label TXPs’ functional types of test peptide sequence. |

**Appendix Note 1: Additional notes on “The proof of Theorem 1”**

**Proof.** $\boldsymbol{\forall}\mathbf{P}_{\boldsymbol{1}}^{\left( d \right)}\boldsymbol{,}\mathbf{P}_{\boldsymbol{2}}^{\left( d \right)}\boldsymbol{\in}\mathbb{R}^{s^{\left( d \right)}\times l}$, we have

$$\left\| \nabla f\left( \mathbf{P}_{\boldsymbol{1}}^{\left( d \right)} \right)-\nabla f\left( \mathbf{P}_{\boldsymbol{2}}^{\left( d \right)} \right) \right\|_{F}^{2}$$

=$\left\| {2a}^{\left( d \right)}\mathbf{X}_{\mathrm{tr}}^{\left( d \right)T}\mathbf{X}_{\mathrm{tr}}^{\left( d \right)}\mathbf{P}_{\boldsymbol{1}}^{\left( d \right)}+\mathbf{X}_{\mathrm{tr}}^{\left( d \right)T}\left( \left| \mathbf{E}-\mathbf{Y⨀}\left( \mathbf{X}_{\mathrm{tr}}^{\left( d \right)}\mathbf{P}_{\boldsymbol{1}}^{\left( d \right)} \right) \right|_{\boldsymbol{+}}\mathbf{⨀}\left( \boldsymbol{-}\mathbf{Y} \right) \right)+2\lambda_{1}\mathbf{P}_{\boldsymbol{1}}^{\left( d \right)}\boldsymbol{+}\mu\mathbf{X}_{\mathrm{tr}}^{\left( d \right)T}\mathbf{X}_{\mathrm{tr}}^{\left( d \right)}\mathbf{P}_{\boldsymbol{1}}^{\left( d \right)}-{2a}^{\left( d \right)}\mathbf{X}_{\mathrm{tr}}^{\left( d \right)T}\mathbf{X}_{\mathrm{tr}}^{\left( d \right)}\mathbf{P}_{\boldsymbol{2}}^{\left( d \right)}-\mathbf{X}_{\mathrm{tr}}^{\left( d \right)T}\left( \left| \mathbf{E}-\mathbf{Y⨀}\left( \mathbf{X}_{\mathrm{tr}}^{\left( d \right)}\mathbf{P}_{\boldsymbol{1}}^{\left( d \right)} \right) \right|_{\boldsymbol{+}}\mathbf{⨀}\left( \boldsymbol{-}\mathbf{Y} \right) \right)-2\lambda_{1}\mathbf{P}_{\boldsymbol{2}}^{\left( d \right)}\boldsymbol{-}\mu\mathbf{X}_{\mathrm{tr}}^{\left( d \right)T}\mathbf{X}_{\mathrm{tr}}^{\left( d \right)}\mathbf{P}_{\boldsymbol{1}}^{\left( d \right)} \right\|_{F}^{2}$

=$\left\| \mathbf{X}_{\mathrm{tr}}^{\left( d \right)T}\left( \left( \left| \mathbf{E}-\mathbf{Y⨀}\left( \mathbf{X}_{\mathrm{tr}}^{\left( d \right)}\mathbf{P}_{\boldsymbol{1}}^{\left( d \right)} \right) \right|_{\boldsymbol{+}}\boldsymbol{-}\left| \mathbf{E}-\mathbf{Y⨀}\left( \mathbf{X}_{\mathrm{tr}}^{\left( d \right)}\mathbf{P}_{\boldsymbol{2}}^{\left( d \right)} \right) \right|_{\boldsymbol{+}} \right)\mathbf{⨀}\left( \boldsymbol{-}\mathbf{Y} \right) \right)+{2a}^{\left( d \right)}\mathbf{X}_{\mathrm{tr}}^{\left( d \right)T}\mathbf{X}_{\mathrm{tr}}^{\left( d \right)}\boldsymbol{\Delta}\mathbf{P}^{\left( d \right)}+2\lambda_{1}\boldsymbol{\Delta}\mathbf{P}^{\left( d \right)}\boldsymbol{+}\mu\mathbf{X}_{\mathrm{tr}}^{\left( d \right)T}\mathbf{X}_{\mathrm{tr}}^{\left( d \right)}\boldsymbol{\Delta}\mathbf{P}^{\left( d \right)} \right\|_{F}^{2}$

=$\left\| \mathbf{X}_{\mathrm{tr}}^{\left( d \right)T}\left( \left( \left| \mathbf{E}-\mathbf{Y⨀}\left( \mathbf{X}_{\mathrm{tr}}^{\left( d \right)}\mathbf{P}_{\boldsymbol{1}}^{\left( d \right)} \right) \right|_{\boldsymbol{+}}\boldsymbol{-}\left| \mathbf{E}-\mathbf{Y⨀}\left( \mathbf{X}_{\mathrm{tr}}^{\left( d \right)}\mathbf{P}_{\boldsymbol{2}}^{\left( d \right)} \right) \right|_{\boldsymbol{+}} \right)\mathbf{⨀}\left( \boldsymbol{-}\mathbf{Y} \right) \right)+\left( \left( {2a}^{\left( d \right)}+\mu\right)\mathbf{X}_{\mathrm{tr}}^{\left( d \right)T}\mathbf{X}_{\mathrm{tr}}^{\left( d \right)}+2\lambda_{1}\mathbf{I} \right)\boldsymbol{\Delta}\mathbf{P}^{\left( d \right)} \right\|_{F}^{2}$ (23)

where $\triangle\mathbf{P}^{\left( d \right)}\boldsymbol{=}\mathbf{P}_{\boldsymbol{1}}^{\left( d \right)}\boldsymbol{-}\mathbf{P}_{\boldsymbol{2}}^{\left( d \right)}$**.** According to the Lemma1 and Lemma2 in [5], the Eq.(23) can be transformed into

$$\left\| \nabla f\left( \mathbf{P}_{\boldsymbol{1}}^{\left( d \right)} \right)-\nabla f\left( \mathbf{P}_{\boldsymbol{2}}^{\left( d \right)} \right) \right\|_{F}^{2}$$

$$\leq2\left\| \mathbf{X}_{\mathrm{tr}}^{\left( d \right)T}\left( \left( \left| \mathbf{E}-\mathbf{Y⨀}\left( \mathbf{X}_{\mathrm{tr}}^{\left( d \right)}\mathbf{P}_{\boldsymbol{1}}^{\left( d \right)} \right) \right|_{\boldsymbol{+}}\boldsymbol{-}\left| \mathbf{E}-\mathbf{Y⨀}\left( \mathbf{X}_{\mathrm{tr}}^{\left( d \right)}\mathbf{P}_{\boldsymbol{2}}^{\left( d \right)} \right) \right|_{\boldsymbol{+}} \right)\mathbf{⨀}\left( \boldsymbol{-}\mathbf{Y} \right) \right) \right\|_{F}^{2}+2\left\| \left( \left( {2a}^{\left( d \right)}+\mu\right)\mathbf{X}_{\mathrm{tr}}^{\left( d \right)T}\mathbf{X}_{\mathrm{tr}}^{\left( d \right)}+2\lambda_{1}\mathbf{I} \right)\boldsymbol{\Delta}\mathbf{P}^{\left( d \right)} \right\|_{F}^{2}$$

$$\leq2\left\| \mathbf{X}_{\mathrm{tr}}^{\left( d \right)T} \right\|_{F}^{2}\left\| \left| \mathbf{E}-\mathbf{Y⨀}\left( \mathbf{X}_{\mathrm{tr}}^{\left( d \right)}\mathbf{P}_{\boldsymbol{1}}^{\left( d \right)} \right) \right|_{\boldsymbol{+}}\boldsymbol{-}\left| \mathbf{E}-\mathbf{Y⨀}\left( \mathbf{X}_{\mathrm{tr}}^{\left( d \right)}\mathbf{P}_{\boldsymbol{2}}^{\left( d \right)} \right) \right|_{\boldsymbol{+}} \right\|_{F}^{2}+2\left\| \left( {2a}^{\left( d \right)}+\mu\right)\mathbf{X}_{\mathrm{tr}}^{\left( d \right)T}\mathbf{X}_{\mathrm{tr}}^{\left( d \right)}+2\lambda_{1}\mathbf{I} \right\|_{F}^{2}\left\| \boldsymbol{\Delta}\mathbf{P}^{\left( d \right)} \right\|_{F}^{2}$$

$$\leq2\left\| \mathbf{X}_{\mathrm{tr}}^{\left( d \right)} \right\|_{F}^{2}\left\| -\mathbf{Y⨀}\left( \mathbf{X}_{\mathrm{tr}}^{\left( d \right)}\boldsymbol{\Delta}\mathbf{P}^{\left( d \right)} \right) \right\|_{F}^{2}+2\left\| \left( {2a}^{\left( d \right)}+\mu\right)\mathbf{X}_{\mathrm{tr}}^{\left( d \right)T}\mathbf{X}_{\mathrm{tr}}^{\left( d \right)}+2\lambda_{1}\mathbf{I} \right\|_{F}^{2}\left\| \boldsymbol{\Delta}\mathbf{P}^{\left( d \right)} \right\|_{F}^{2}$$

$$\leq2\left\| \mathbf{X}_{\mathrm{tr}}^{\left( d \right)} \right\|_{F}^{2}\left\| \mathbf{X}_{\mathrm{tr}}^{\left( d \right)} \right\|_{F}^{2}\left\| \boldsymbol{\Delta}\mathbf{P}^{\left( d \right)} \right\|_{F}^{2}+2\left\| \left( {2a}^{\left( d \right)}+\mu\right)\mathbf{X}_{\mathrm{tr}}^{\left( d \right)T}\mathbf{X}_{\mathrm{tr}}^{\left( d \right)}+2\lambda_{1}\mathbf{I} \right\|_{F}^{2}\left\| \boldsymbol{\Delta}\mathbf{P}^{\left( d \right)} \right\|_{F}^{2}$$

$=\left( 2\left( \left\| \mathbf{X}_{\mathrm{tr}}^{\left( d \right)} \right\|_{F}^{2} \right)^{2}+2\left\| \left( {2a}^{\left( d \right)}+\mu\right)\mathbf{X}_{\mathrm{tr}}^{\left( d \right)T}\mathbf{X}_{\mathrm{tr}}^{\left( d \right)}+2\lambda_{1}\mathbf{I} \right\|_{F}^{2} \right)\left\| \boldsymbol{\Delta}\mathbf{P}^{\left( d \right)} \right\|_{F}^{2}$ (24)

**References**

[1] W. Hu, D. Tao, W. Zhang, Y. Xie, and Y. Yang, “The Twist Tensor Nuclear Norm for Video Completion,” *IEEE transactions on neural networks and learning systems,* vol. 28, no. 12, pp. 2961-2973, Dec, 2017.

[2] Y. Chen, X. Xiao, and Y. Zhou, “Multi-view subspace clustering via simultaneously learning the representation tensor and affinity matrix,” *Pattern Recognition,* vol. 106, pp. 107441, 2020.

[3] Y. Nesterov, “Smooth minimization of non-smooth functions,” *Mathematical Programming,* vol. 103, no. 1, pp. 127-152, May, 2005.

[4] J. F. Cai, E. J. Candès, and Z. W. Shen, “A Singular Value Thresholding Algorithm for Matrix Completion,” *Siam Journal on Optimization,* vol. 20, no. 4, pp. 1956-1982, 2010.

[5] G. Wu, R. Zheng, Y. Tian, and D. Liu, “Joint ranking SVM and binary relevance with robust low-rank learning for multi-label classification,” *Neural Networks,* vol. 122, pp. 24-39, 2020.
